# OsFeSOD3 Functions as an Enzymatic Component of the PEP Complex, Bifunctionally Regulating Chloroplastic ROS Metabolism and Chloroplast Biogenesis in Rice

**DOI:** 10.1111/pbi.70508

**Published:** 2025-12-17

**Authors:** Deok Hyun Seo, Jiwoong Jung, Geupil Jang

**Affiliations:** ^1^ School of Biological Sciences and Technology Chonnam National University Gwangju Republic of Korea

**Keywords:** chloroplast biogenesis, chloroplastic ROS, OsFeSOD3, plastid‐encoded RNA polymerase

## Abstract

Chloroplasts are essential organelles responsible for photosynthesis, providing energy and metabolic intermediates required for plant growth and productivity. Chloroplast development is highly sensitive to environmental stresses such as drought, and this sensitivity is closely associated with growth inhibition and yield reduction under stress conditions. However, the molecular mechanisms governing this process remain largely elusive. In this study, we demonstrate that chloroplastic ROS metabolism plays a pivotal role in modulating chloroplast development in response to abiotic stress, and we identify *OsFeSOD3*, which encodes a chloroplast‐localised iron superoxide dismutase, as a key regulator of this process. Time‐lapse visualisation of cellular ROS dynamics and characterisation of *OsFeSOD3*‐overexpressing rice showed that *OsFeSOD3‐*mediated chloroplastic ROS metabolism is tightly associated with cytoplasmic ROS accumulation under stress conditions, and that overexpression of *OsFeSOD3* is sufficient to enhance rice stress tolerance by reducing cellular ROS accumulation. Furthermore, agronomic trait analyses over 2 years of cultivation revealed that *OsFeSOD3*‐overexpressing rice exhibits a 33%–42% increase in grain yield under drought conditions compared with wild‐type plants, highlighting *OsFeSOD3* as a promising genetic target for developing stress‐tolerant, high‐yielding crops. Moreover, phenotypic and molecular characterisation of *OsFeSOD3* knock‐out mutants indicates that OsFeSOD3 functions as a PEP‐complex component regulating chloroplast biogenesis in rice, a role further supported by its direct interaction with other PEP‐complex proteins. Taken together, our findings suggest that *OsFeSOD3* serves as a bifunctional regulator that coordinates chloroplastic ROS metabolism and chloroplast biogenesis in rice.

## 1 Introduction

Abiotic stresses such as drought, salinity, and oxidative conditions are major constraints on global agricultural productivity. These environmental factors disrupt diverse physiological processes in plants, including membrane fluidity, enzymatic activity, and osmotic balance, thereby destabilising cellular homeostasis and limiting growth and yield (Ahuja et al. 2010; Chang et al. 2024; Parry et al. 2002; van Veen et al. 2025). In response to these stresses, cellular ROS levels increase markedly and mediate many of the associated physiological perturbations (Liu et al. 2025; Mani et al. 2025; Mittler et al. 2022; Morales and Munné‐Bosch 2016; Xiong et al. 2018). The central role of ROS in plant stress responses and tolerance has been further demonstrated by molecular genetic studies of cytoplasmic ROS‐scavenging genes. Overexpression of ascorbate peroxidase (*APX*, such as *AtAPX1* and *OsAPXs*) and catalase (*CAT*, such as *AtCAT2* and *OsCATc*) reduced ROS accumulation under abiotic stress conditions, enhancing tolerance to drought, salinity, and cold, whereas knock‐out mutants exhibited increased cellular ROS levels and decreased tolerance to abiotic stress (Davletova et al. 2005; Liao et al. 2023; Lu et al. 2007; Ono et al. 2021; Sato et al. 2011; Zhang et al. 2013). It has been suggested that a variety of metabolic processes such as mitochondrial respiration, chloroplastic photosynthesis, and peroxisomal photorespiration contribute to the increase and accumulation of cellular ROS under stress conditions (Apel and Hirt 2004; Hamanaka and Chandel 2010; Li and Kim 2022; Wang, Liu, et al. 2024; Yamashita et al. 2008; Yang and Lian 2020; Yu et al. 2019; Zhu et al. 2018), yet many questions including the relationship between organelle ROS and plant stress response remain elusive.

Chloroplasts are specialised plastids that function as the primary sites of photosynthesis in plants, and their development is highly sensitive to abiotic stresses. Stress‐induced alterations in chloroplast development include a range of structural and functional disruptions, such as chlorophyll degradation, disorganisation of thylakoid and grana architecture, and a consequent decline in photosynthetic efficiency (Chauhan et al. 2023; Iqbal and Munir 2024; Isgandarova et al. 2024; Lu et al. 2023; Manaa et al. 2019; Zou et al. 2022). Studies on chloroplast‐localised antioxidant enzymes have suggested that ROS levels in chloroplasts are deeply involved in the stress‐induced modulation of chloroplast development. For instance, transgenic rice overexpressing chloroplastic *OsCu/ZnSOD* exhibited reduced chlorophyll degradation under salt stress (Guan et al. 2017). Similarly, heterologous expression of *PsCu/ZnSOD* in tobacco and *SaCu/ZnSOD* in *Arabidopsis* alleviated stress‐induced chlorophyll loss and preserved photosynthetic efficiency under various abiotic stress conditions, including drought, cold, and oxidative stress (Gupta, Heinen, et al. 1993; Gupta, Webb, et al. 1993; Li et al. 2017). Plastid‐targeted expression of flavodoxin also decreased chloroplastic ROS accumulation and maintained chloroplast ultrastructure under abiotic stress conditions (Mayta et al. 2018; Tognetti et al. 2006). The involvement of chloroplast ROS in stress‐induced modulation of chloroplast development is further supported by the relationship between gerontoplast formation and chloroplastic ROS accumulation. During senescence, ROS accumulate in chloroplasts, which develop into gerontoplasts, and this developmental transition is marked by the disassembly of thylakoid membranes, degradation of chlorophyll, and accumulation of plastoglobuli, lipid‐rich bodies (Barry 2009; Domínguez and Cejudo 2021; Golczyk et al. 2014; Mayta et al. 2018; McRae and Thompson 1983; Zentgraf et al. 2022). Furthermore, accumulating evidence suggests that chloroplastic ROS metabolism is involved not only in the developmental modulation of chloroplasts but also in plant tolerance to abiotic stresses by affecting cellular ROS levels (Guan et al. 2017; Maruta et al. 2010; Tognetti et al. 2006; Zhuang et al. 2021). Although the dynamics of cellular ROS at the chloroplast–cytoplasm interface remain largely unknown, these findings suggest that plant tolerance to abiotic stress can be improved by regulating chloroplast ROS metabolism, and that chloroplastic ROS‐scavenging genes may serve as promising targets for developing stress‐tolerant crops.

Chloroplasts possess their own genome of 120–160 kb, which contains 110–130 genes that encode components of the photosynthetic apparatus, including *RbcL*, *PsaA*, and *PsaB* (Cauz‐Santos 2025; Daniell et al. 2021). Transcription of the chloroplast genes is a key step determining chloroplast biogenesis, and a key player in this process is the Plastid‐Encoded RNA Polymerase (PEP) (Chang et al. 2017; Garcia et al. 2008; He et al. 2018; Pfalz and Pfannschmidt 2013; Wu, Mu, et al. 2024). PEP assembles into a functional transcriptional machinery, known as the PEP complex, through interactions with a variety of nuclear‐encoded components, particularly PEP‐associated proteins (PAPs) (Pfalz et al. 2015; Steiner et al. 2011; Wang, Wang, et al. 2024). Recent evidence suggests that the formation and stabilisation of the PEP complex is a critical step that determines PEP activity and chloroplast biogenesis (do Prado et al. 2024; Jeon et al. 2012; Kindgren et al. 2012; Seo et al. 2024; Steiner et al. 2011; Vergara‐Cruces et al. 2024; Wang, Liu, et al. 2016; Wu, Mu, et al. 2024). Interestingly, previous studies have shown that the transcription of PEP‐dependent chloroplast genes, such as *RbcL*, *PsaA*, and *PsaB*, is suppressed under stress conditions (Boroujerdnia et al. 2020; Danilova et al. 2018; Lu et al. 2023). Considering the tight relationship between ROS and chloroplast development, these findings suggest that chloroplastic ROS metabolism is closely linked to PEP‐dependent chloroplast biogenesis, potentially through shared regulatory factors.

Here, we propose that *OsFeSOD3*, a chloroplast‐localised ROS‐scavenging gene, plays a bifunctional role in regulating both chloroplastic ROS metabolism and chloroplast biogenesis. This conclusion is supported by our experimental findings, which show that *OsFeSOD3* overexpression and knock‐out mutation produce clearly opposing effects on these processes. Additionally, our study reveals that chloroplastic ROS directly contributes to the increase and accumulation of cellular ROS under abiotic stress conditions, and that suppression of chloroplastic ROS accumulation through *OsFeSOD3* overexpression is sufficient to reduce cellular ROS levels and enhance rice tolerance to abiotic stresses such as drought, salinity, and oxidative stress.

## 2 Results

### 2.1 The Increase in Cellular ROS Levels Under Abiotic Stress Is Primarily Driven by ROS Accumulation in Chloroplasts

**FIGURE 1:**
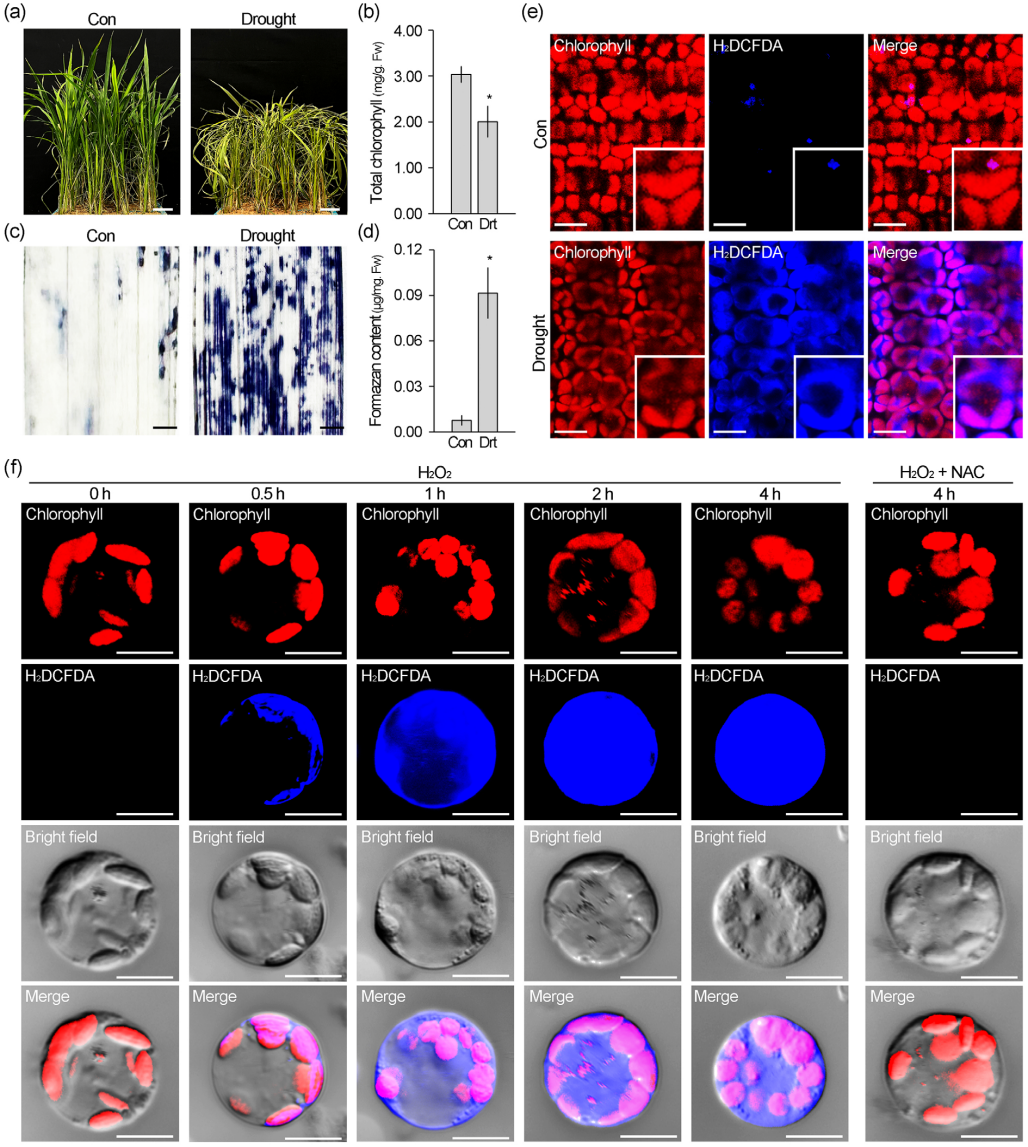
Chloroplastic ROS accumulation is tightly associated with cellular ROS accumulation under abiotic stress. (a, b) Images showing the morphology of 5‐week‐old rice plants under normal (Con) and after 2 days of drought stress (Drought, Drt) (a), and quantification of total chlorophyll content in these plants (b). (c, d) NBT staining results indicating cellular ROS levels (c) and quantification of ROS levels (d) in these plants. (e) Visualisation of chloroplastic ROS accumulation using H_2_DCFDA staining in the wild‐type plants under control and drought stress conditions. (f) Time‐course analysis of H_2_DCFDA fluorescence signals in protoplasts in response to oxidative stress. Protoplasts isolated from 1‐week‐old rice seedlings were treated with 2 mM H_2_O_2_ alone or co‐treated with 2 mM H_2_O_2_ and 1 mM NAC, and incubated for the indicated time. Red and blue fluorescence correspond to chlorophyll autofluorescence and H_2_DCFDA signals, respectively. Quantification data represent the means of seven biological replicates. Error bars indicate SD. Asterisks denote statistically significant differences between the corresponding samples and their controls (*p < 0.01, two‐tailed *t*‐test). Scale bars = 5 cm in (a), 0.1 cm in (c), 5 μm in (e) and 10 μm in (f).

Rice exposed to drought stress exhibited leaf rolling and wilting, as well as leaf yellowing resulting from chlorophyll degradation (Figure 1a,b). NBT staining for ROS visualisation revealed that drought stress markedly increases cellular ROS levels in the drought‐stressed rice, with quantification showing an approximately 12‐fold increase in response to drought stress (Figure 1c,d). To further investigate ROS accumulation at the organelle level, we performed H_2_DCFDA staining (Figure 1e). In the leaves of drought‐untreated rice, chloroplasts exhibited strong chlorophyll autofluorescence and no or only weak H_2_DCFDA fluorescence (blue). By contrast, drought‐stressed rice showed reduced chlorophyll autofluorescence and markedly increased H_2_DCFDA signals in chloroplasts, and this pattern was similarly observed in rice exposed to salinity and oxidative stress conditions (Figures S1 and S2). These observations suggest that abiotic stress induces a strong accumulation of ROS in chloroplasts, which is closely associated with the overall increase in cellular ROS levels. This finding was further supported by time‐course imaging using a protoplast system, which revealed dynamic changes in chloroplastic and cytoplasmic ROS levels in response to oxidative stress (Figure 1f). In untreated control protoplasts, no H_2_DCFDA signals were detected. After 30 min of H_2_O_2_ treatment, H_2_DCFDA signals appeared only in chloroplasts with no detectable signals in other cellular compartments. By 1 h, the chloroplastic H_2_DCFDA signals intensified to near‐maximal levels, and H_2_DCFDA signals also began to emerge in the cytoplasm. As treatment time increased, cytoplasmic signals also intensified, following a pattern similar to that observed in chloroplasts. Consequently, in protoplasts treated with H_2_O_2_ for 2 or 4 h, H_2_DCFDA signals reached maximal intensity throughout the cells, including both chloroplasts and cytoplasm. Notably, cytoplasmic H_2_DCFDA signals were never observed prior to those in chloroplasts, and protoplasts co‐treated with H_2_O_2_ and the antioxidant N‐acetyl‐*L*‐cysteine (NAC) did not exhibit H_2_DCFDA signals in either chloroplasts or cytoplasm even after 4 h unlike those treated with H_2_O_2_ alone. These findings indicate that the elevation of cellular ROS levels under stress conditions is predominantly dependent on ROS accumulation in chloroplasts, thereby implicating chloroplastic ROS metabolism as a crucial process in regulating plant tolerance to abiotic stress.

### 2.2 Chloroplast Localization and Stress‐Dependent Regulation of OsFeSOD3

**FIGURE 2:**
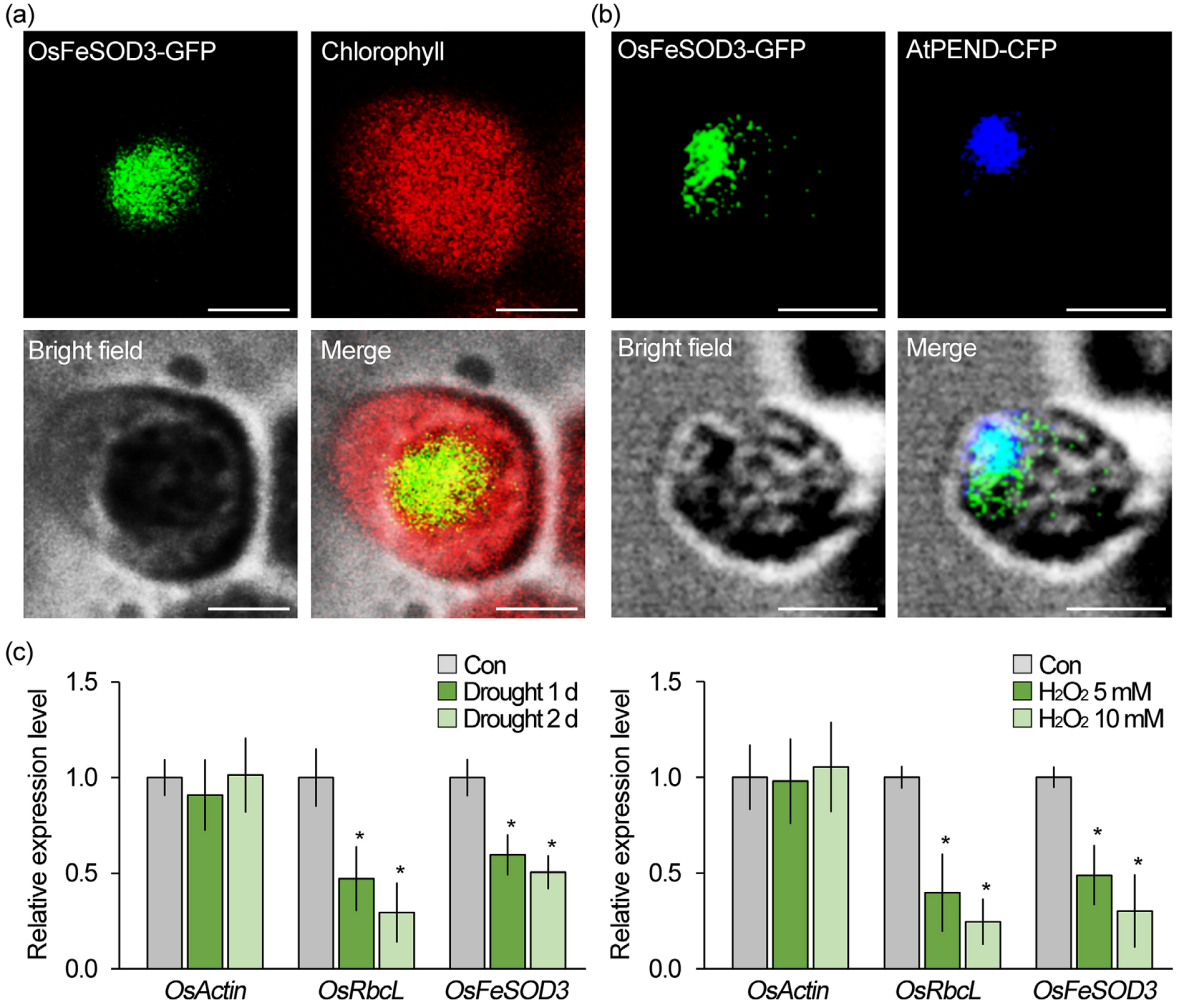
Characterisation of *OsFeSOD3* expression and localization. (a) Visualisation of OsFeSOD3 localization using GFP fusion and protoplast expression system. The OsFeSOD3‐GFP fusion proteins were transiently expressed by introducing 35S::OsFeSOD3‐GFP plasmids into protoplasts isolated from 1‐week‐old rice. (b) Colocalization analysis of OsFeSOD3 with AtPEND, a chloroplast nucleoid‐associated protein. Protoplasts isolated from 4‐week‐old *Arabidopsis* were co‐transformed with 35S::OsFeSOD3‐GFP and 35S::AtPEND‐CFP plasmids, and the fluorescent signals were observed. Green, blue and red fluorescence correspond to GFP, CFP and chlorophyll autofluorescence signals, respectively. (c) Quantitative RT‐PCR analysis shows that *OsFeSOD3* expression is downregulated in response to abiotic stress such as drought (left) and H_2_O_2_ treatment (right). Total mRNA was extracted from 1‐week‐old wild‐type rice exposed to drought stress for 1 and 2 days, or treated with 5 and 10 mM H_2_O_2_ for 1 day. *OsTUB2* was used as a reference gene to normalise the RT‐qPCR results. RT‐qPCR data are the means of 9 replicates (3 biological × 3 technical). Error bars indicate SD. Asterisks indicate statistically significant differences between the corresponding samples and their controls (*p < 0.01, two‐tailed *t*‐test). Scale bars = 2 μm.

To investigate the role of chloroplastic ROS in rice drought stress tolerance, we identified OsFeSOD3, a putative chloroplast‐localised iron superoxide dismutase that shares high amino acid sequence homology with *Arabidopsis* FeSOD3 (Figure S3), a well‐characterised enzyme known for its chloroplast localization and SOD activity (Lee et al. 2019). We confirmed the localization of OsFeSOD3 proteins in chloroplasts using GFP fusion and fluorescence signal visualisation (Figure 2a). Notably, the observation of OsFeSOD3‐GFP signals as dot‐like structures within chloroplasts suggests that OsFeSOD3 proteins specifically localise to chloroplast nucleoids. This was further supported by colocalization analysis with nucleoid‐specific PEND (AtPEND‐CFP) and OsFeSOD3 proteins (OsFeSOD3‐GFP). In chloroplasts, the green fluorescent signals of OsFeSOD3‐GFP overlapped with the blue fluorescent signals of AtPEND‐CFP, indicating that OsFeSOD3 proteins are localised to chloroplast nucleoids (Figure 2b). Furthermore, we found that *OsFeSOD3* expression is tightly regulated by abiotic stress. It was downregulated under drought and oxidative stress, with the reduction becoming more evident as drought duration increased and H_2_O_2_ concentrations rose (Figure 2c). Its expression pattern closely resembled that of *OsRbcL*, a well‐established molecular indicator of stress whose transcription is strongly suppressed by abiotic stress (Jaskulak et al. 2018; Winicov 1996), while *OsActin* remained unaffected. These findings support the conclusion that *OsFeSOD3* expression is suppressed under stress conditions.

### 2.3 *OsFeSOD3* Overexpression Suppresses Chloroplast‐To‐Gerontoplast Transition

**FIGURE 3:**
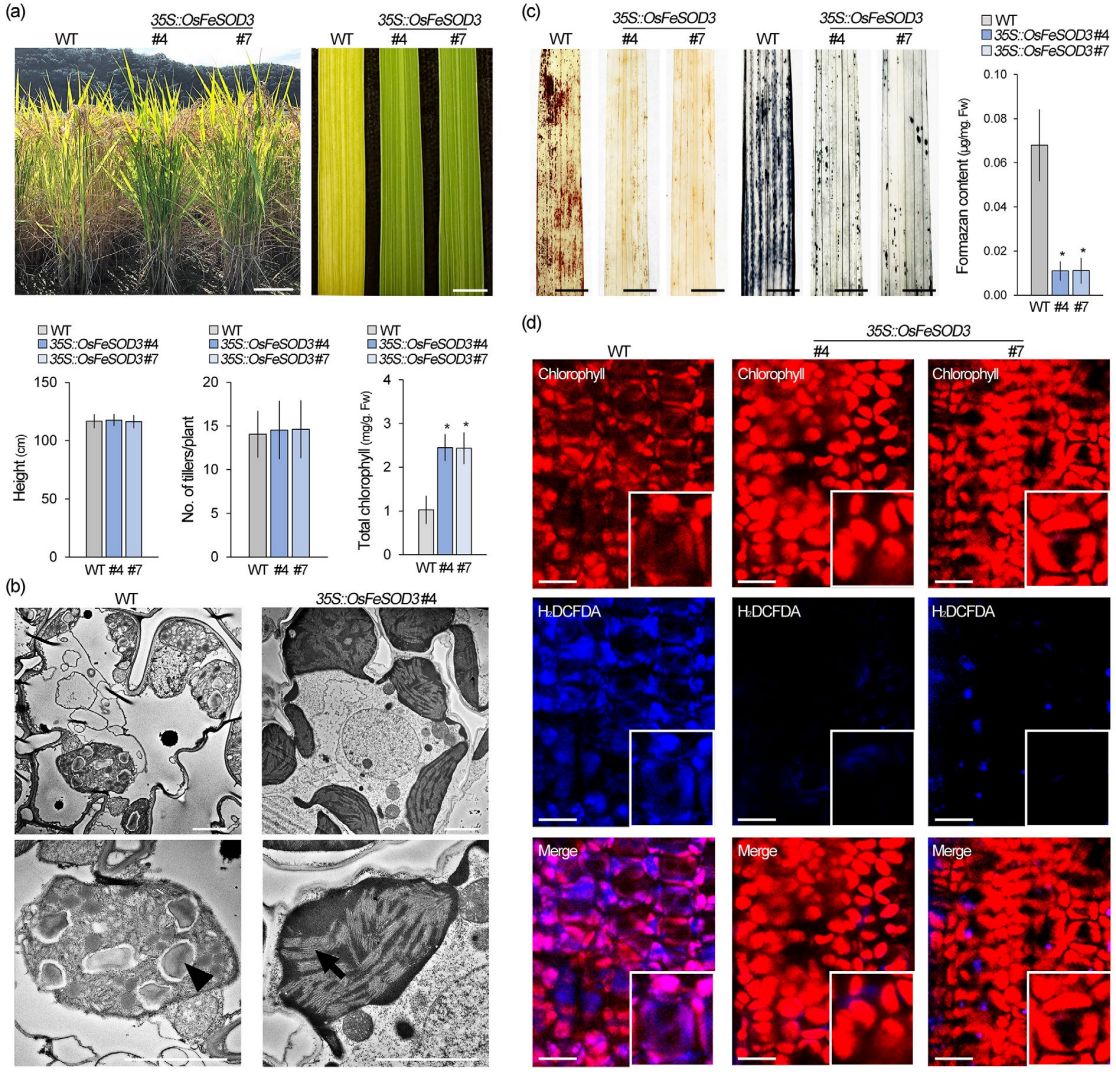
Gerontoplast formation is suppressed by *OsFeSOD3* overexpression. (a) Morphology of wild‐type (WT) and *OsFeSOD3*‐overexpressing rice at the harvest stage (top), and quantification of plant height, tiller number, and chlorophyll content in these plants (bottom, *n* > 34). #4 and 7 indicate two independent T_3_ lines of *35S::OsFeSOD3* plants. (b) Transmission electron microscopic images showing ultrastructure of chloroplasts formed in these plants. The arrow and arrowhead indicate thylakoid membranes and starch granules, respectively. (c) Analysis of cellular ROS levels in these plants using DAB and NBT staining. The graph shows the quantification of NBT staining intensity by measuring formazan content. (d) Visualisation of ROS accumulation in chloroplasts using H_2_DCFDA staining in these plants. Red and blue fluorescence correspond to chlorophyll autofluorescence and H_2_DCFDA signals, respectively. Quantification data represent the means of three biological replicates. Error bars indicate SD. Asterisks denote statistically significant differences between the corresponding samples and their controls (*p < 0.01, two‐tailed *t*‐test). Scale bars = 20 cm (left) and 1 cm (right) in (a), 2 μm in (b), 1 cm in (c) and 5 μm in (d).

To investigate the role of *OsFeSOD3* in chloroplastic ROS metabolism, we generated *OsFeSOD3*‐overexpressing rice and selected two independent transgenic lines with significantly elevated *OsFeSOD3* expression levels (Figure S4). In vegetative stage rice, the overexpression of *OsFeSOD3* did not affect phenotypic traits such as plant height, tiller number, or leaf colour (Figure S5). However, at the harvest stage, *OsFeSOD3*‐overexpressing plants exhibited a noticeable delay in leaf senescence compared to wild‐type plants (Figure 3a). While 5‐month‐old wild‐type rice typically displayed a senescent phenotype characterised by yellowing and pale green leaves with reduced chlorophyll content, *OsFeSOD3*‐overexpressing rice at the same developmental stage maintained greener leaves with higher chlorophyll levels. Transmission electron microscopy (TEM) of plastid morphology revealed that wild‐type plastids had the characteristics of gerontoplasts, senesced chloroplasts characterised by swollen thylakoids, disorganised grana stacks, and large plastoglobuli accumulation. In contrast, plastids in *OsFeSOD3*‐overexpressing rice displayed features of unsenesced chloroplasts, with well‐developed thylakoids and densely packed grana stacks (Figure 3b). These findings suggest that *OsFeSOD3* overexpression delays leaf senescence by inhibiting the developmental transition from chloroplasts to gerontoplasts.

Since ROS is a crucial factor controlling gerontoplast formation and senescence (Biswal and Raval 2003; Botticella et al. 2024; Muñoz and Munné‐Bosch 2018; Parida et al. 1978; Rogers and Munné‐Bosch 2016), we hypothesised that the delayed senescence observed in *OsFeSOD3*‐overexpressing plants may result from altered cellular ROS levels. To test this, we analysed ROS levels using DAB and NBT staining. *OsFeSOD3*‐overexpressing plants exhibited significantly reduced staining intensity compared to wild‐type rice, indicating lower cellular ROS levels (Figure 3c). Further analysis using H_2_DCFDA staining revealed that the reduction in ROS levels is closely associated with decreased chloroplastic ROS, due to the overexpression of *OsFeSOD3*, which functions in scavenging chloroplastic ROS. As expected, chloroplastic ROS signals were not detected both in 3‐month‐old wild‐type and *OsFeSOD3*‐overexpressing rice at the vegetative stage (Figure S6). However, 5‐month‐old wild‐type plants at the harvest stage exhibited strong H_2_DCFDA signals in chloroplasts, whereas only weak signals were detected in *OsFeSOD3*‐overexpressing rice at the same developmental stage (Figure 3d). These findings provide mechanistic insight into how *OsFeSOD3* overexpression suppresses the chloroplast‐to‐gerontoplast transition by attenuating chloroplastic ROS accumulation.

### 2.4 *OsFeSOD3* Overexpression Enhances Drought Stress Tolerance in Rice

**FIGURE 4:**
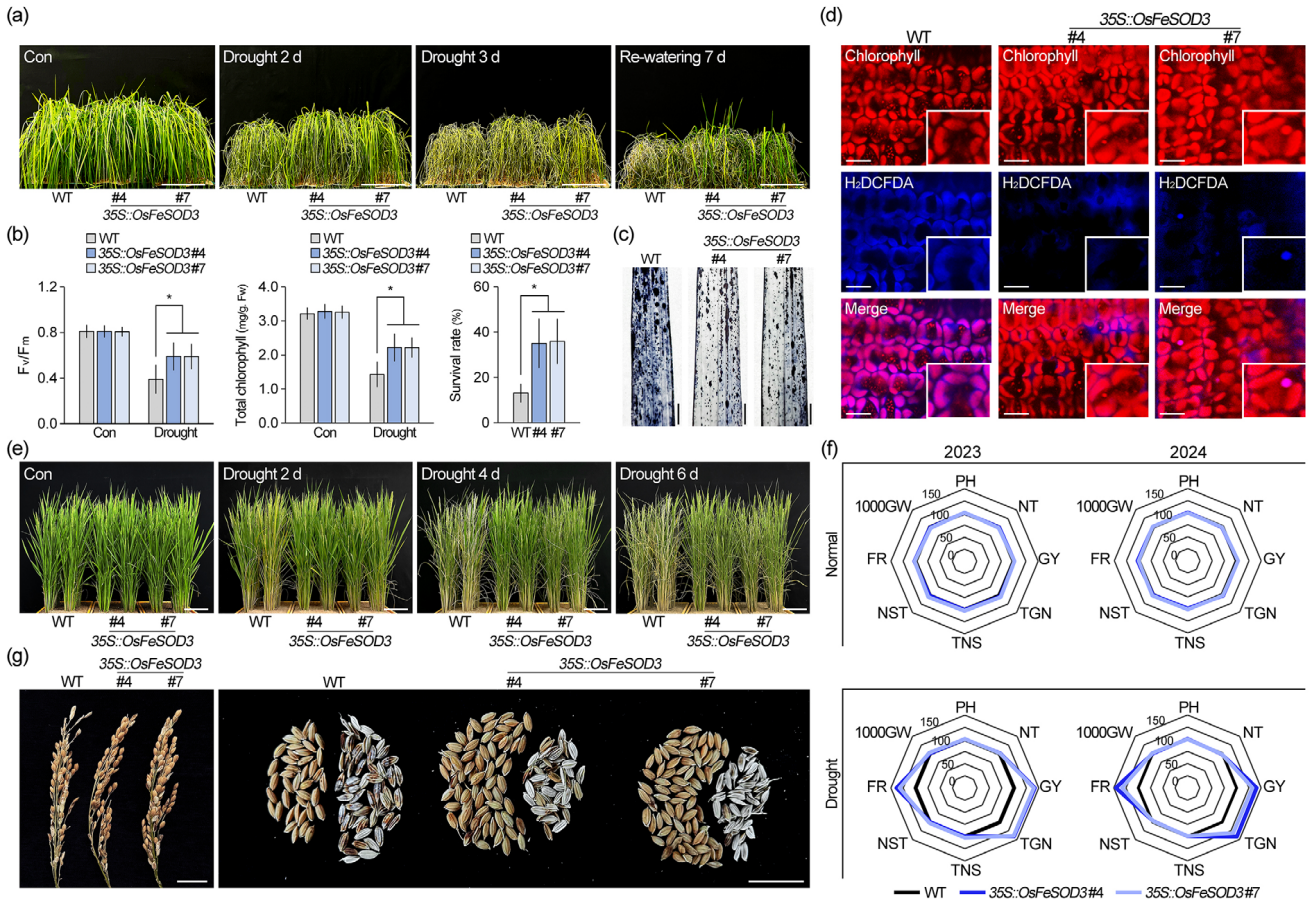
Overexpression of *OsFeSOD3* enhances drought stress tolerance and increases grain yield. (a) Morphology of 4‐week‐old wild‐type (WT) and *OsFeSOD3*‐overexpressing (*35S::OsFeSOD3*) rice treated with drought stress. Plants were exposed to dehydration conditions for 3 days, followed by re‐watering for 7 days. #4 and 7 indicate two independent T_3_ lines of *35S::OsFeSOD3* plants. (b) Photosynthetic efficiency (left) and chlorophyll content (middle) measured in the wild‐type and *35S::OsFeSOD3* plants exposed to drought stress for 2 days (*n* > 142). The survival rate (right) was calculated by dividing the number of plants that survived after re‐watering by the total number of plants tested (*n* > 480). (c) NBT‐stained images of rice leaves after 2 days of drought treatment. (d) Visualisation of chloroplastic ROS accumulation using H_2_DCFDA staining in these plants. (e) Images of 4‐month‐old wild‐type and *OsFeSOD3*‐overexpressing rice under dehydration conditions. (f) Growth and yield of wild‐type and *OsFeSOD3*‐overexpressing rice cultivated under normal and drought stress conditions. 2023 (T_3_) and 2024 (T_4_) indicate the cultivation years. Each point represents the percentage relative to the mean of wild‐type rice, set to 100%. PH, plant height; NT, number of tillers per plant; GY, grain yield per plant; TGN, total grain number per plant; TNS, total number of spikelets per plant; NST, number of spikelets per tiller; FR, grain filling rate; 1000GW, 1000‐grain weight (*n* > 21, for each year). (g) Images of harvested panicles (left) and grains (right) showing similar spikelet numbers per plant but clear differences in grain filling rates between wild‐type and *OsFeSOD3*‐overexpressing rice. Quantification data of photosynthesis efficiency, chlorophyll content and survival rate represent the means of six biological replicates. Error bars indicate SD. Asterisks indicate statistically significant differences between the corresponding samples and their control (*p < 0.01, two‐tailed *t*‐test). Scale bars = 20 cm in (a) and (e), 0.5 cm in (c), 5 μm in (d) and 2 cm in (g).

Since chloroplastic ROS metabolism regulates cellular ROS levels, which play a critical role in plant responses and tolerance to drought stress (Møller et al. 2007; Tognetti et al. 2006), we hypothesized that overexpression of *OsFeSOD3* could enhance drought tolerance in rice. To test this, 1‐month‐old wild‐type and *OsFeSOD3*‐overexpressing transgenic rice were exposed to dehydration conditions, and their phenotypic and physiological responses were monitored over time (Figure 4a,b). After 2 days of drought stress, both wild‐type and *OsFeSOD3*‐overexpressing rice showed symptoms of drought stress, such as chlorosis, leaf rolling, and wilting (Figure 4a). However, these symptoms in *OsFeSOD3*‐overexpressing rice were less severe compared to wild‐type plants, as confirmed by measurements of photosynthetic activity and chlorophyll content (Figure 4b). Drought stress reduced photosynthetic activity and chlorophyll content by 52% and 56% in wild‐type plants, whereas *OsFeSOD3*‐overexpressing rice showed only 27% and 32% reductions, respectively. These findings suggest that *OsFeSOD3* overexpression enhances drought stress tolerance in rice, and the improved tolerance is also evident in survival rates (Figure 4b). After re‐watering and 10 days of recovery under normal conditions, the survival rate of *OsFeSOD3*‐overexpressing rice was 2.7 times higher than that of wild‐type plants. NBT staining showed that stress‐induced cellular ROS accumulation is reduced by *OsFeSOD3* overexpression, which was supported by H_2_O_2_ quantification (Figure 4c; Figure S7). Visualisation of H_2_DCFDA fluorescence further revealed that *OsFeSOD3*‐overexpressing lines display substantially lower chloroplastic ROS signals than wild‐type plants under stress conditions (Figure 4d; Figure S8). These results indicate that *OsFeSOD3* overexpression suppresses chloroplastic ROS accumulation, which in turn reduces cellular ROS levels and enhances drought stress tolerance.

To assess the effects of *OsFeSOD3* overexpression on agronomic traits under drought stress, field‐grown wild‐type and *35S::OsFeSOD3* rice at the reproductive stage were subjected to drought conditions. A comprehensive set of growth and yield parameters, including plant height, tiller number, heading date, spikelet number, grain filling rate, grain number, 1000‐grain weight, and grain yield, was evaluated over 2 years of cultivation (2023 and 2024) (Figure 4e–g; Table S1). As expected, *OsFeSOD3*‐overexpressing rice exhibited enhanced drought tolerance at the reproductive stage, consistent with the improved stress tolerance observed during the vegetative stage (Figure S9). More importantly, we found that the enhanced drought tolerance conferred by *OsFeSOD3* overexpression led to a significant increase in grain yield under drought conditions. While no yield differences were observed between wild‐type and *35S::OsFeSOD3* plants under normal growth conditions, the transgenic rice produced approximately 33%–42% more grain yield than wild‐type plants under drought stress. Grain yield per plant ranged from 9.41 to 9.59 g in wild‐type rice, but from 12.71 to 13.52 g in *OsFeSOD3*‐overexpressing rice. Notably, the increase closely paralleled the improvement in grain filling rate and grain number per plant. Grain filling rate and grain number per plant were 57.00%–62.69% and 728.66–796.18 in *OsFeSOD3*‐overexpressing rice, compared with 42.77%–43.83% and 545.40–554.01 in wild‐type plants, corresponding to increases of 32%–47% and 32%–44%, respectively. Plant height, tiller number, heading date, spikelet number per tiller and 1000‐grain weight were 104.19–106.23 cm, 12.61–12.71, 107–108 DAS, 100.73–103.34 and 17.17–17.91 g, respectively, in *OsFeSOD3*‐overexpressing rice, and these parameters were nearly identical to those of wild‐type plants. These findings indicate that *OsFeSOD3* overexpression improves grain yield under drought stress, primarily through enhanced grain filling rate and increased grain number.

### 2.5 Loss of *OsFeSOD3* Function Disrupts Chloroplast Biogenesis, Leading to an Albino Phenotype

**FIGURE 5:**
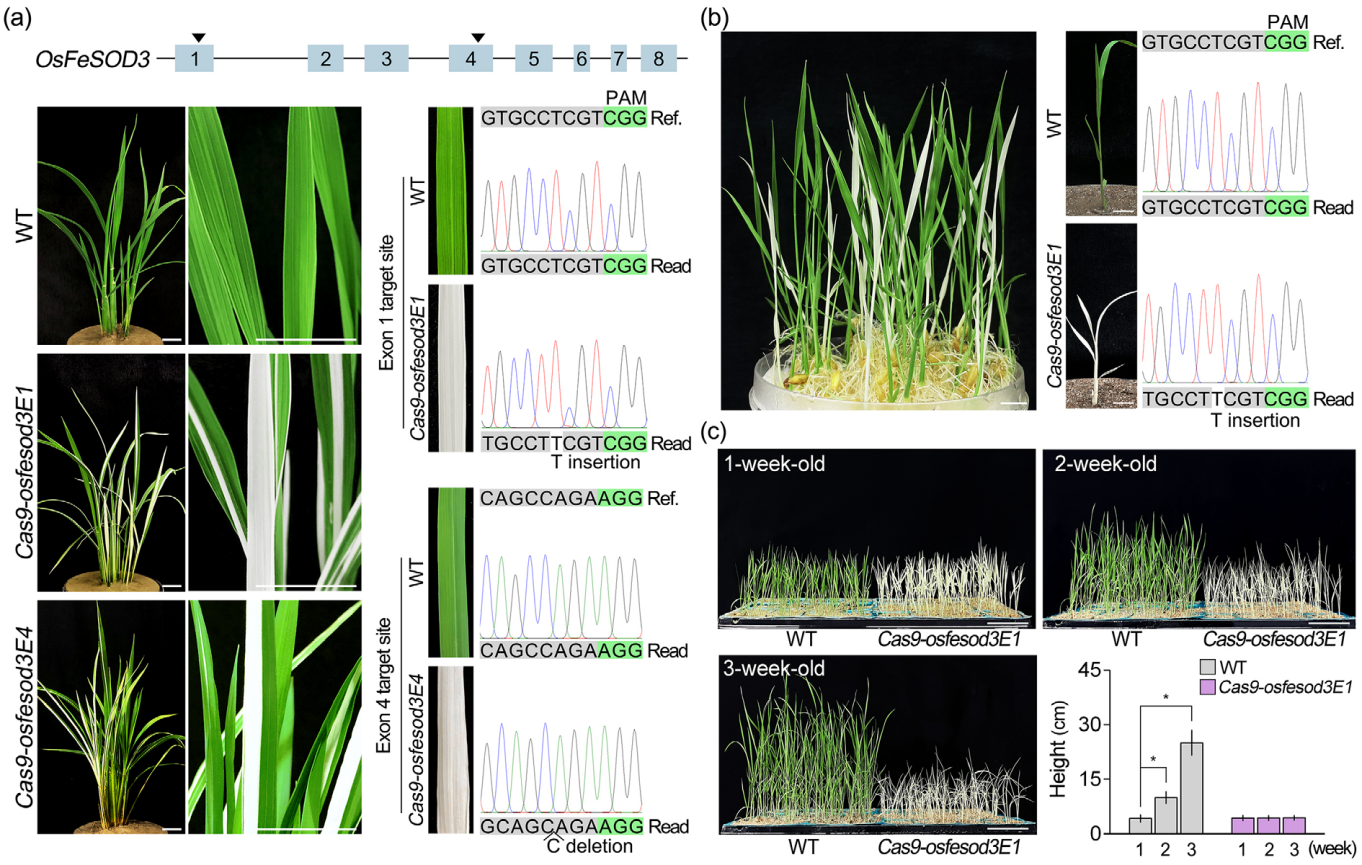
Knock‐out mutation of *OsFeSOD3* causes an albino phenotype in rice. (a) Abnormal albino phenotype of T_0_ *Cas9‐osfesod3* transgenic rice (*Cas9‐osfesod3E1* and *Cas9‐osfesod3E4*). The schematic indicates the guide RNA target sites for mutations at *OsFeSOD3* exon 1 and exon 4 (arrowheads), and DNA sequencing results showing that the albino leaf tissue carries a homozygous mutation at the *OsFeSOD3* target sites. (b) Segregation of the *Cas9‐osfesod3E1* albino phenotype in the T_1_ generation, and DNA sequencing results showing that the T_1_ *Cas9‐osfesod3E1* rice with an albino phenotype carries a homozygous mutation at the *OsFeSOD3* target site. Green boxes indicate the protospacer adjacent motif (PAM) sequence, and letters point homozygous mutations. (c) Growth of WT and *OsFeSOD3* homozygous mutant (*Cas9‐osfesod3E1*) plants over time, and quantification results (*n* > 168). Quantification data are the means of six biological replicates. Error bars indicate SD. Asterisks indicate statistically significant differences between the samples and controls. (*p < 0.01, two‐tailed *t*‐test). Scale bar = 2 cm in (a), 1 cm in (b) and 4 cm in (c).

To further explore the role of *OsFeSOD3*, we generated *OsFeSOD3* knock‐out rice mutants, *Cas9‐osfesod3E1* and *Cas9‐osfesod3E4*, in which exon 1 or exon 4 of *OsFeSOD3* was disrupted using the CRISPR/Cas9 system (Figure 5a). In the T_0_ generation, both *Cas9‐osfesod3E1* and *Cas9‐osfesod3E4* transgenic plants displayed an albino phenotype, characterised by green‐and‐white‐striped or completely white leaves. DNA sequencing revealed that the albino tissues carried a homozygous mutation at the target *OsFeSOD3* site (*Cas9‐osfesod3E1*; T insertion, *Cas9‐osfesod3E4*; C deletion). Characterisation of T_1_ progeny further demonstrated the link between the albino phenotype and the *OsFeSOD3* knock‐out mutation (Figure 5b). Approximately 25% of the T_1_ *Cas9‐osfesod3E1* plants exhibited the albino phenotype (green: albino = 215: 71), and *χ* ^2^ analysis confirmed a Mendelian 3:1 segregation ratio (*χ* ^2^ = 0.005, *p* > 0.05). As expected, all albino T_1_ *Cas9‐osfesod3E1* plants were homozygous for the *OsFeSOD3* mutation, whereas no homozygous mutations were detected in green T_1_ plants. Chlorophyll quantification in leaf blades and sheaths indicated that the albino phenotype of *OsFeSOD3* knock‐out mutants results from a severe reduction in chlorophyll levels (Figure S10). When we monitored growth and leaf colour for 3 weeks in wild‐type and *OsFeSOD3* homozygous mutant plants, the albino phenotype did not recover over time. Although growth was comparable during the first week, the homozygous mutants showed growth arrest thereafter, whereas wild‐type plants continued to grow (Figure 5c). These results indicate that *OsFeSOD*3 is essential for leaf greening and normal growth in rice.

**FIGURE 6:**
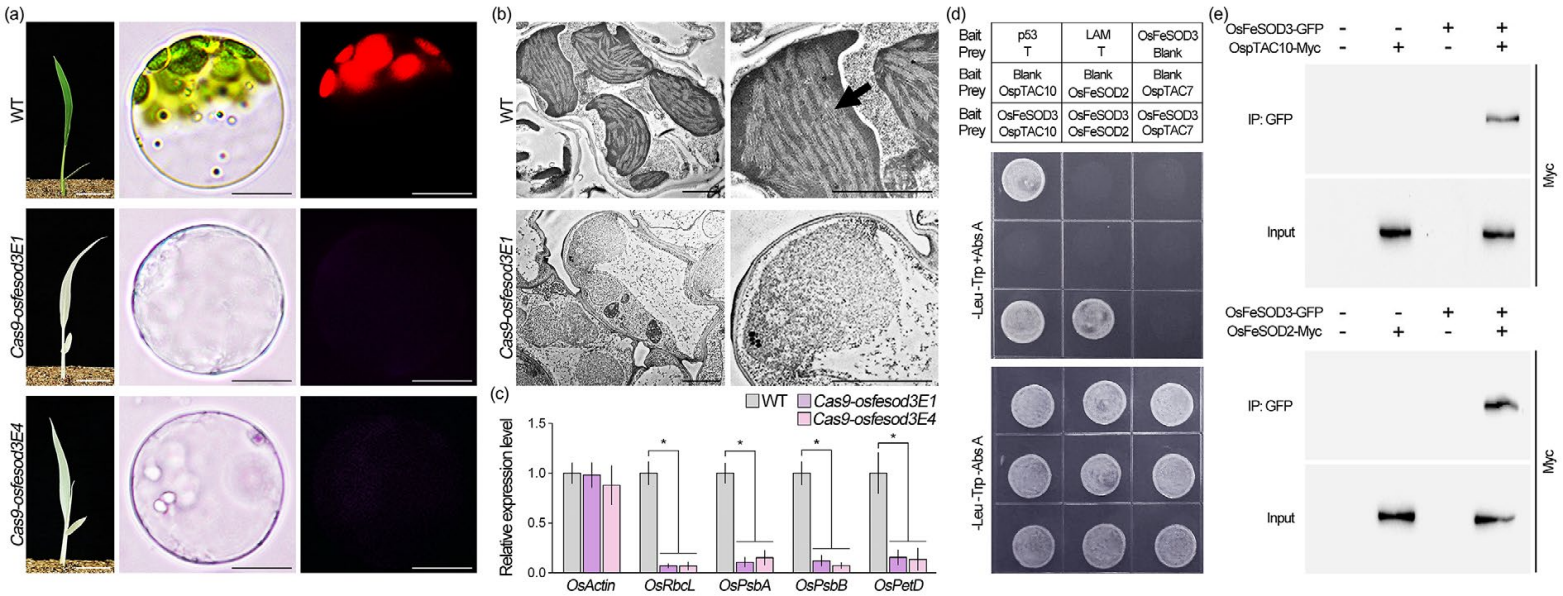
Knock‐out mutation of *OsFeSOD3* disrupts chloroplast biogenesis in rice. (a) Images showing the severe impairment of chloroplast biogenesis in *osfesod3* knock‐out mutants (*Cas9‐osfesod3E1* and *E4*). The left panel shows 1‐week‐old plant images, while the middle and right panels display bright‐field and fluorescence images of protoplasts isolated from these plants, respectively. (b, c) TEM images of chloroplast ultrastructure (b) and RT‐qPCR analysis of chloroplast expression levels (c) in these plants. The arrow indicates thylakoid membranes. *OsTUB2* served as a reference gene for normalisation. (d) Yeast two‐hybrid assay testing interactions of OsFeSOD3 with rice PEP complex components such as OspTAC10, OsFeSOD2, and OspTAC7. Co‐transformation with the p53 bait and T prey plasmids was used for a positive control and co‐transformation with the LAM bait and T prey plasmids served as a negative control. Blank indicates empty bait or prey plasmids. ‐Leu ‐Trp + Abs A indicates double dropout (DDO) medium containing Aureobasidin A (Abs A) for analysing interactions with OsFeSOD3, while ‐Leu ‐Trp—Abs A indicates DDO medium without Abs A for validating yeast transformation and equal dropping. (e) Co‐immunoprecipitation (Co‐IP) analysis of OsFeSOD3 interactions with OspTAC10 and OsFeSOD2. Total proteins were extracted from 1‐week‐old rice protoplasts co‐transformed with 35S::OsFeSOD3‐GFP and either 35S::OspTAC10‐Myc or 35S::OsFeSOD2‐Myc. Immunoprecipitation was performed using anti‐GFP antibody, followed by immunoblotting with anti‐Myc antibody. Data represent the means of 9 replicates (3 biological × 3 technical). Error bars indicate SD, and asterisks indicate statistically significant differences between the corresponding samples and their controls (*p < 0.01, two‐tailed *t*‐test). Scale bars = 2 cm (left) and 10 μm (middle and right) in (a) and 2 μm in (b).

Given the role of *OsFeSOD3* in leaf greening, we hypothesised that its knock‐out would disrupt chloroplast development. To test this, we isolated protoplasts from wild‐type and *OsFeSOD3* mutant leaves and examined plastid morphology (Figure 6a). While wild‐type protoplasts contained chloroplasts, green plastids exhibiting chlorophyll autofluorescence under UV light, *OsFeSOD3* knock‐out protoplasts lacked green plastids and showed no detectable chlorophyll autofluorescence. Additionally, transmission electron microscopy (TEM) imaging revealed that plastids in wild‐type plants contained well‐organised thylakoids and densely arranged grana with proper thylakoid stacking. In contrast, the plastids of *OsFeSOD3* mutant rice lacked observable thylakoid and grana structures (Figure 6b). These findings suggest that chloroplast biogenesis is disrupted in *OsFeSOD3* mutant rice.

PEP, the major RNA polymerase in chloroplasts, assembles into a functional transcriptional machinery, the PEP complex, through protein–protein interactions among its components (Chang et al. 2017; do Prado et al. 2024; He et al. 2018; Vergara‐Cruces et al. 2024; Wang, Wang, et al. 2024; Wu, Mu, et al. 2024). Transcription of chloroplast genes by the PEP complex constitutes a key step in chloroplast biogenesis (Ahrens et al. 2025; Pfalz et al. 2006; Pfalz and Pfannschmidt 2013; Seo et al. 2024; Yagi et al. 2012). To explore the role of *OsFeSOD3* in rice chloroplast biogenesis, we analysed PEP‐dependent transcription in *OsFeSOD3* knock‐out rice. Transcript levels of PEP‐dependent chloroplast genes, including *OsRbcL*, *OsPsbA*, *OsPsbB* and *OsPetD*, were markedly reduced (Figure 6c), indicating that *OsFeSOD3* is essential for PEP‐dependent transcription. To further investigate its molecular function, we used AlphaFold‐Multimer (AFM), an AI‐based tool for predicting protein–protein interactions and three‐dimensional structures (Abramson et al. 2024; Guo et al. 2025; Homma et al. 2023, 2024), to evaluate potential interactions between OsFeSOD3 and 14 rice PEP‐complex components (Table S2; Figure S11). AFM predicted high‐confidence interactions of OsFeSOD3 with OsFeSOD2 and OspTAC10, with scores exceeding those of well‐characterised pairs such as OsIAA1–OsARF25 and OsJAZ9–OsSLR1 (Li et al. 2022; Um et al. 2018; Wu, Cao, et al. 2024). Yeast two‐hybrid and GST pull‐down assays validated interactions of OsFeSOD3 with OsFeSOD2 and OspTAC10, whereas no interaction was detected with OspTAC7, consistent with the low‐confidence prediction by AFM (Figure 6d; Figure S12). Similarly, co‐immunoprecipitation also detected OsFeSOD3–OsFeSOD2 and OsFeSOD3–OspTAC10 interactions (Figure 6e). Together, these findings suggest that OsFeSOD3 functions as a component of the rice PEP complex, explaining the severe disruption of PEP‐dependent transcription and chloroplast biogenesis in *OsFeSOD3* knock‐out plants. Additionally, *OsFeSOD3* is expressed specifically in leaves and is positively regulated by light (Figure S13), a key environmental signal for chloroplast biogenesis, which further underscores its critical role in this process.

### 2.6 Disruption of Chloroplastic ROS Scavenging by the *OsFeSOD3* Mutation Causes Cellular ROS Accumulation

**FIGURE 7:**
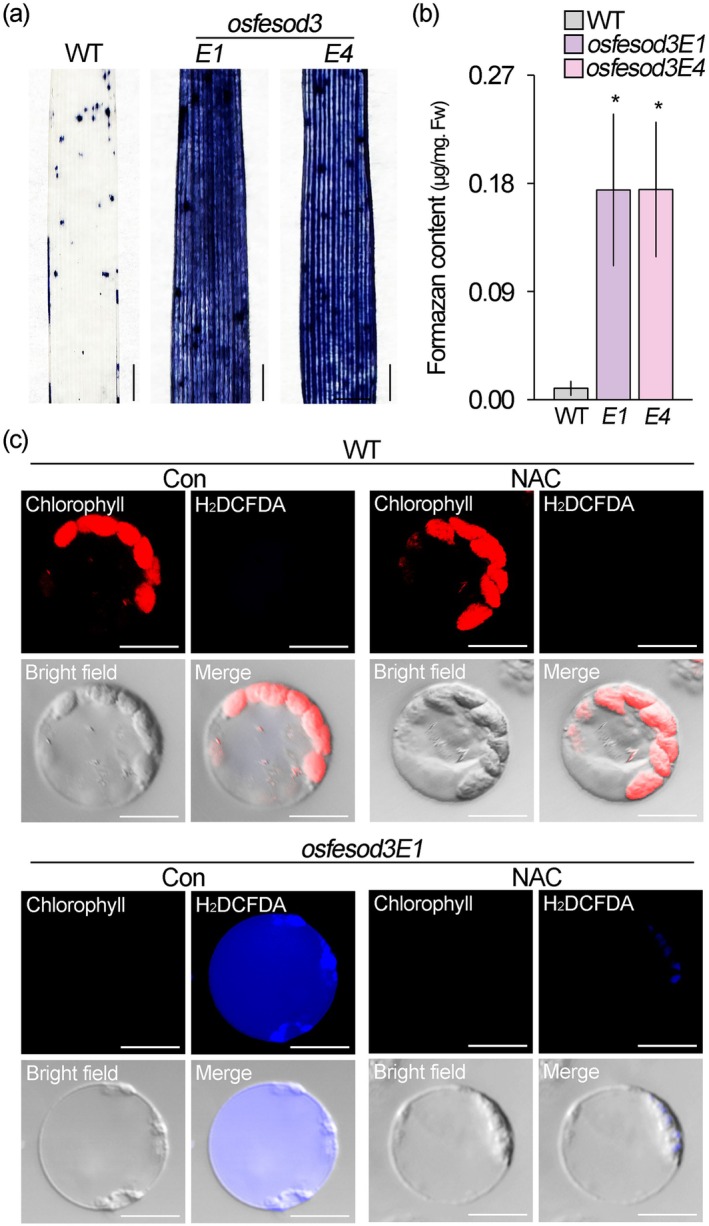
Mutation of *OsFeSOD3* increases cellular ROS levels, with predominant ROS accumulation in plastids. (a, b) Analysis of cellular ROS levels in 1‐week‐old wild‐type (WT) and *osfesod3* knock‐out mutant (*osfesod3E1* and *osfesod3E4*) plants using NBT staining (a) and quantification of NBT staining intensity by measuring formazan content (b). (c) H_2_DCFDA fluorescence signals in wild‐type and *osfesod3E1* protoplasts incubated ‐untreated (Con) or ‐treated with 1 mM NAC (NAC). Red and blue fluorescence correspond to chlorophyll autofluorescence and H_2_DCFDA signals, respectively. Quantification data represent the means of seven biological replicates. Error bars indicate SD. Asterisks denote statistically significant differences between the corresponding samples and their controls (*p < 0.01, two‐tailed *t*‐test). Scale bars = 0.2 cm in (a) and 10 μm in (c).

To explore the effect of *OsFeSOD3* knock‐out mutation on ROS metabolism, we visualised ROS levels in *OsFeSOD3* knock‐out mutants grown under normal growth conditions using NBT staining. *OsFeSOD3* knock‐out mutant plants exhibited markedly higher NBT staining than wild‐type plants (Figure 7a). Quantification of NBT staining intensity revealed an approximately 18‐fold increase in ROS levels in *OsFeSOD3* knock‐out mutant plants compared to wild‐type plants (Figure 7b), indicating that mutation of *OsFeSOD3* significantly increases cellular ROS levels in rice. To further investigate this at the organelle level, we isolated protoplasts from wild‐type and *OsFeSOD3* knock‐out mutant plants and visualised chlorophyll and H_2_DCFDA fluorescence signals. Even under non‐stress conditions, *osfesod3* protoplasts exhibited H_2_DCFDA fluorescence in both the cytoplasm and plastids, with particularly intense signals in plastids and widespread cytoplasmic distribution (Figure 7c). In contrast, chlorophyll autofluorescence was not detected, and the H_2_DCFDA fluorescence signals disappeared upon NAC treatment. This pattern was the opposite of that observed in wild‐type protoplasts, which displayed strong chlorophyll autofluorescence and no detectable H_2_DCFDA fluorescence, supporting the role of *OsFeSOD3* in chloroplastic ROS metabolism. Taken together, our experimental results suggest that OsFeSOD3 is a bifunctional regulator controlling chloroplastic ROS metabolism and chloroplast biogenesis in rice (Figure S14).

## 3 Discussion

Cellular ROS levels play a crucial role in plant responses and tolerance to abiotic stress, and growing evidence suggests that organelle‐specific ROS metabolism is involved in cellular ROS levels under abiotic stress conditions (Guan et al. 2017; Joo et al. 2014; Kim et al. 2021; Li and Kim 2022; Li et al. 2017; Liao et al. 2023; McKersie et al. 1996; Wang et al. 2004; Zhang et al. 2013; Zhu et al. 2018). In this study, we showed that abiotic stress markedly increases ROS accumulation in chloroplasts, and *OsFeSOD3* is deeply involved in this process. Visualisation of H_2_DCFDA fluorescence and RT‐qPCR analysis revealed that wild‐type rice subjected to drought, salt or oxidative stress displayed strong H_2_DCFDA signals in chloroplasts and significantly reduced transcript levels of *OsFeSOD3*. Unlike wild‐type plants, *OsFeSOD3*‐overexpression lines exhibited substantially lower chloroplastic ROS signals than wild‐type plants under stress conditions, indicating the essential role of *OsFeSOD3* in chloroplastic ROS metabolism under stress conditions.

Importantly, time‐course analysis of organelle‐specific ROS provided further insight into the spatiotemporal dynamics of ROS accumulation under stress, as well as into the role of *OsFeSOD3* in modulating these dynamics. In wild‐type rice, no ROS signals were detected in any cellular organelle under non‐stress conditions. In response to stress, ROS signals first appeared in chloroplasts, gradually accumulating and reaching peak levels as stress duration increased. Cytoplasmic ROS signals were observed only after chloroplastic ROS had reached their peak. In contrast, the *OsFeSOD3* knock‐out mutant exhibited ROS accumulation in both chloroplasts and the cytoplasm even under non‐stress conditions, with chloroplastic ROS levels consistently higher than those in the cytoplasm. These findings suggest that the dynamics of cellular ROS accumulation under stress conditions largely originate from chloroplasts. The observation that overexpression of *OsFeSOD3* alters these dynamics, delaying the appearance of cytoplasmic ROS signals (Figure S15), further supports this idea and provides insight into why *OsFeSOD3* overexpression enhances drought tolerance. In this study, we showed that overexpression of *OsFeSOD3* enhances rice tolerance to drought stress, consistent with a study by Wang et al. (2021) (Wang et al. 2021). Given its role in chloroplastic ROS scavenging and cellular ROS dynamics, these results suggest that by promoting chloroplastic ROS scavenging, *OsFeSOD3* overexpression suppresses cytoplasmic ROS accumulation, thereby conferring improved drought stress tolerance. These findings are further supported by additional results showing that *OsFeSOD3*‐overexpressing rice also exhibits improved tolerance to various abiotic stresses, including salinity and oxidative stress (Figures S16 and S17), and by previous studies reporting the role of chloroplastic ROS metabolism in plant responses to abiotic stresses. For example, overexpression of the chloroplast ROS scavenging gene *OsCu/ZnSOD* reduced cellular ROS and improved stress tolerance, whereas its knock‐down had the opposite effect (Guan et al. 2017; Lu et al. 2010). Similarly, heterologous expression of *PsCu/ZnSOD* enhanced oxidative and cold stress tolerance in tobacco, and stabilisation of chloroplastic Cu/ZnSOD via *EGY3* overexpression improved salt tolerance in *Arabidopsis* (Gupta, Heinen, et al. 1993; Gupta, Webb, et al. 1993; Zhuang et al. 2021).

Abiotic stresses are major constraints on global crop productivity. Climate models predict that their frequency and intensity will increase with global warming, potentially worsening crop yield losses worldwide (Gebrechorkos et al. 2025; Tran et al. 2025). In this study, our agronomic trait analysis showed that *OsFeSOD3*‐overexpressing transgenic rice exhibits approximately 33%–42% increase in grain yield under drought conditions. ROS metabolism plays a central role in plant tolerance to abiotic stresses, including drought, salinity, and oxidative stress, and OsFeSOD3 is a key regulator of chloroplastic and cellular ROS levels, thereby conferring improved stress tolerance. Furthermore, the strong conservation of FeSOD3 across the plant kingdom, including major crops such as barley, maize, wheat, tobacco, and tomato, as well as early‐diverging land plants such as moss, suggests its broad applicability and potential as a molecular target for enhancing tolerance to multiple abiotic stresses and improving grain yield in rice and other major crops.

Characterisation of *OsFeSOD3* knock‐out mutants, which completely lack chloroplasts, further indicates that *OsFeSOD3* is essential for chloroplast biogenesis, extending its function beyond chloroplastic ROS metabolism. Transcription of chloroplast genes is a critical step in chloroplast biogenesis, primarily driven by plastid‐encoded RNA polymerase (PEP) (Ahrens et al. 2025; Allison et al. 1996; Börner et al. 2015; Legen et al. 2002). PEP assembles into a functional transcriptional complex (PEP complex) through interactions with other component proteins such as PEP‐associated proteins (PAPs), and increasing evidence strongly suggests that proper assembly of the PEP complex is crucial for PEP's transcriptional activity and, consequently, for chloroplast biogenesis (Chang et al. 2017; Garcia et al. 2008; Pfalz et al. 2006; Pfalz and Pfannschmidt 2013; Wang, Wang, et al. 2024; Yu et al. 2013). Supporting this, knock‐out mutants of *PEP* or *PAP* genes exhibit defects in chloroplast biogenesis (Allison et al. 1996; De Santis‐Maciossek et al. 1999; He et al. 2018; Seo et al. 2024; Wang, Wang, et al. 2016; Yu et al. 2018). Notably, recent high‐resolution cryo‐EM studies of the PEP complex in tobacco and mustard provided important mechanistic insights into the PEP complex. These analyses suggested that the assembly of the PEP complex requires the cooperative action of more than ten PAPs, which stabilise the complex through PEP–PAP and PAP–PAP interactions. Among these PAPs, FeSOD3/PAP4 was identified as one of the essential PEP‐complex components that integrates into the complex and contributes to its stability and function (do Prado et al. 2024; Vergara‐Cruces et al. 2024; Wu, Mu, et al. 2024). These findings suggest that OsFeSOD3 plays a similar role in rice. In this study, yeast two‐hybrid, GST pull‐down, and co‐immunoprecipitation assays revealed that OsFeSOD3 directly interacts with rice PEP‐complex proteins, including OspTAC10/OsPAP3 and OsFeSOD2/OsPAP9. Combined with previous reports of FeSOD3 interactions with pTAC10/PAP3 and FeSOD2/PAP9 in *Arabidopsis*, as well as OsFeSOD3 interaction with OsTrxZ/OsPAP10 in rice (Chang et al. 2017; Myouga et al. 2008; Wang et al. 2021), these results indicate that OsFeSOD3 functions as a PEP‐complex component for rice chloroplast biogenesis. This provides a mechanistic explanation for the disrupted chloroplast biogenesis and albino phenotype observed in *OsFeSOD3* knock‐out mutants. Furthermore, subcellular localization analysis corroborates this model by showing that OsFeSOD3 is specifically localised to the chloroplast nucleoid where the PEP complex acts. Taken together, we propose that *OsFeSOD3*, acting both as a chloroplastic ROS scavenger and as a component of the PEP complex, plays a bifunctional role in coordinating chloroplastic ROS metabolism and chloroplast biogenesis.

## 4 Experimental Procedures

### 4.1 Plant Material, Growth and Stress Treatment

Rice ( *Oryza sativa* L.) cultivar Dongjin was used as the wild‐type (WT) control. Seeds were surface‐sterilised with 2.5% (v/v) sodium hypochlorite for 1 h and then washed thoroughly with sterile distilled water. The sterilised seeds were germinated on half‐strength Murashige and Skoog (½ MS) solid medium for 3 days in a growth chamber (16 h light/8 h light/dark, 32°C), and seedlings were transferred to soil and grown under the same conditions. To apply drought treatment to rice at the vegetative stage, 15 rice seedlings were grown for 4 weeks in each cavity of a 4 × 4 soil‐filled plug tray (16 cavities, 6 × 6 × 5 cm each) under normal growth chamber conditions, and then were exposed to dehydration conditions. Survival rates were calculated as the ratio of surviving plants to the total number of tested plants. For oxidative stress treatment, sterilised seeds were germinated and grown on ½ MS solid medium containing 10 mM H_2_O_2_ for 2 weeks in growth chamber conditions.

### 4.2 Generation of *OsFeSOD3* Knock‐Out and Overexpression Transgenic Rice

The CRISPR/Cas9 system was used to generate knock‐out mutant rice lines of *OsFeSOD3* (*Cas9‐osfesod3E1* and *Cas9‐osfesod3E4*). Guide RNA sequences targeting exon 1 and exon 4 were designed using the CRISPR‐P 2.0 website (http://crispr.hzau.edu.cn/cgi‐bin/CRISPR2/CRISPR). These guide RNAs were inserted into the *B*saI site of the pRGEB31 vector using T4 ligase‐mediated DNA ligation (New England Biolabs) to construct the Cas9‐osfesod3E1 and Cas9‐osfesod3E4 plasmids. To generate *OsFeSOD3*‐overexpressing transgenic rice, pMDC‐35S::OsFeSOD3 plasmids were constructed using a Gateway system (Invitrogen). To do this, *OsFeSOD3* cDNA was amplified by reverse transcription PCR and introduced into the pDONR221 vector using a BP reaction (Invitrogen). The pMDC‐35S::OsFeSOD3 plasmids were generated by introducing the OsFeSOD3 cDNA in the entry clone (pENTRY‐OsFeSOD3) into the pMDC32 vectors using an LR reaction (Invitrogen). Cas9‐osfesod3E1/E4 and pMDC‐35S::OsFeSOD3 plasmids were introduced into embryogenic calli derived from mature seeds of *Oryza sativa* cv. Dongjin via Agrobacterium‐mediated transformation, as described by Hiei et al. (1994) (Hiei et al. 1994). Briefly, calli were co‐cultivated with Agrobacterium strain LBA4404 harbouring the respective plasmids. T_0_ transformants regenerated from the transformed calli were initially selected through antibiotic screening and further confirmed by DNA sequencing and transgene expression analysis. Primer sequences used for vector construction are listed in Table S3.

### 4.3 Protoplast Isolation and Transient Expression

For subcellular localization, rice protoplast isolation and transient expression were used as previously described by Zhang et al. (2011) with slight modifications (Zhang et al. 2011). Briefly, protoplasts were isolated from 1‐week‐old wild‐type rice grown on ½ MS solid medium. Leaf tissues were incubated in an enzyme solution containing 1.5% cellulase RS, 0.75% macerozyme R‐10, 0.6 M mannitol, 10 mM MES (pH 5.7), 10 mM CaCl_2_ in the dark. Following digestion, the protoplasts were washed three times with W5 buffer (154 mM NaCl, 125 mM CaCl_2_, 5 mM KCl and 2 mM MES at pH 5.7) and resuspended in MMG solution (0.4 M mannitol, 15 mM MgCl_2_ and 4 mM MES at pH 5.7). To observe subcellular localization of OsFeSOD3, protoplasts were transformed with 35S::OsFeSOD3‐GFP plasmids and incubated for 12 h in the dark. GFP fluorescence was detected using a confocal microscope (STED, Leica). For colocalization analysis of OsFeSOD3 and AtPEND, protoplasts were isolated from 4‐week‐old *Arabidopsis thaliana* grown in a growth chamber (16 h/8 h = light/dark, 23°C), as previously described by Yoo et al. (2007) (Yoo et al. 2007). The protoplasts were co‐transformed with 35S::OsFeSOD3‐GFP and 35S::AtPEND‐CFP and incubated for 12 h in the dark. Chlorophyll autofluorescence, GFP and CFP fluorescent signals were detected by confocal microscope (STED, Leica). Chlorophyll autofluorescence and CFP fluorescence were excited at 458 nm. GFP fluorescence was excited at 488 nm. Fluorescence was detected at 650 to 700 nm for chlorophyll autofluorescence, 460 to 500 nm for CFP, and 505 to 530 nm for GFP. The laser intensity was approximately 10%, and the gain was below 350.

### 4.4 Visualisation and Quantification of ROS Levels

Diaminobenzidine (DAB) and nitroblue tetrazolium (NBT) staining were performed to visualise the cellular ROS levels as previously described by Park et al. (2024) with slight modifications (Park et al. 2024). For DAB and NBT staining, leaves collected from the indicated plants were incubated in DAB staining solution (1 mg/mL DAB in 10 mM Na_2_HPO_4_ [pH 3.0]) or NBT staining solution (0.1% [w/v] nitroblue tetrazolium in 10 mM sodium azide and 50 mM potassium phosphate buffer [pH 6.4]) at room temperature overnight in the dark. After staining, the leaves were incubated in a bleaching solution (ethanol:glycerol:acetic acid at a ratio of 3:1:1, v/v/v) to remove chlorophyll. NBT staining intensity was quantified by measuring the formazan content in the NBT‐stained samples as previously described by Grellet Bournonville and Díaz‐Ricci (2011) with slight modifications (Grellet Bournonville and Díaz‐Ricci 2011). The NBT‐stained samples (0.2 g) were ground with liquid nitrogen, and formazans were extracted using an extraction solution (2 M potassium hydroxide:DMSO at a ratio of 1:1.6, v/v). The absorbance of the extraction solution was measured at 630 nm (Molecular Devices VersaMax Microplate Reader), and formazan content was calculated as previously described by Grellet Bournonville and Díaz‐Ricci (2011) (Grellet Bournonville and Díaz‐Ricci 2011).

To visualise ROS levels at the organelle level, H_2_DCFDA staining was performed as previously described by Ghosh et al. (2017) with slight modifications (Ghosh et al. 2017). A 100 mM stock solution of H_2_DCFDA was prepared in dimethyl sulfoxide (DMSO). Leaves collected from the indicated plants were incubated in 20 μM H_2_DCFDA diluted in sterilised water for 1 h in the dark. Protoplasts were isolated from the indicated plants and stained with 20 μM H_2_DCFDA prepared in MMG solution for 30 min in the dark. H_2_DCFDA fluorescence was excited at 488 nm and detected at 500–570 nm using a confocal microscope.

Hydrogen peroxide (H_2_O_2_) content was quantified as described by Mur et al. (2005) with minor modifications (Mur et al. 2005). Briefly, leaves (0.1 g) from the indicated plants were homogenised in 3 mL of 50 mM phosphate buffer (pH 7.0) at 4°C, and the homogenates were centrifuged at 6000 × *g* for 25 min. One millilitre of the resulting supernatant was mixed with 0.3 mL of 1% (v/v) TiCl_4_ in 10 M HCl, followed by centrifugation at 6000 × *g* for 15 min. The absorbance of the supernatant was then measured at 410 nm using a VersaMax Microplate reader (Molecular Devices).

### 4.5 Measurement of Chlorophyll Content and Photosynthesis Efficiency

For quantification of chlorophyll content, fresh leaves (0.2 g) were collected from the indicated plants and incubated in 10 mL of 80% (v/v) acetone in the dark for 24 h. The incubated samples were centrifuged at 12000 × *g* for 5 min, and the supernatant was diluted 10‐fold using 80% acetone. Absorbance was measured at 646 and 663 nm using a spectrophotometer (VersaMax Microplate Reader, Molecular Devices), and chlorophyll content was calculated according to the method of Wellburn (1994) (Wellburn 1994). To analyse photosynthetic efficiency, the maximum quantum yield of PSII (F_v_/F_m_) was measured as previously described by Shim et al. (2021) (Shim et al. 2021). Leaf samples were dark‐adapted for 1 h using leaf clips to stabilise PSII reaction centers prior to measurement. For each plant, the F_v_/F_m_ values were measured at the top, middle, and base regions of the leaves using a portable fluorometer (Pocket PEA, Hansatech Instruments), and the average was used for analysis.

### 4.6 RNA Extraction and RT‐qPCR Analysis

Total RNA was extracted from the indicated plant using the RNeasy Plant Mini Kit (Qiagen), following the manufacturer's protocol. The first cDNA strand synthesis was performed with 1 μg of total RNA using oligo(dT) primers and GoScript Reverse Transcriptase (Promega). RT‐qPCR was performed using AccuPower GreenStar master mix (Bioneer), and the PCR reaction and fluorescence detections were performed with the CFX Connect Real‐Time PCR Detection System (Bio‐Rad). Three technical replicates of RT‐qPCRs were performed using three biological replicates. Thermal cycling conditions followed the manufacturer's instructions: initial denaturation at 95°C for 5 min, followed by 45 cycles of denaturation at 95°C for 10 s, annealing at 58°C for 10 s, and extension at 72°C for 10 s. Primer sequence information is available in Table S3.

### 4.7 Transmission Electron Microscopy

For the visualisation of chloroplast ultrastructure, ultra‐microsectioning was performed as described previously by Motohashi et al. (2001) with slight modifications (Motohashi et al. 2001). Leaf samples were collected from the indicated plants grown on ½ MS solid medium for 1 week. These samples were fixed for 24 h at room temperature using fixing solution 1 (0.86 M Na‐P [pH 7.2], 1% paraformaldehyde, and 1% glutaraldehyde), and then washed three times with washing solution (0.137 M Na‐P [pH 7.2]). Secondary fixation was performed for 1 h at room temperature using fixing solution 2 (0.86 M Na‐P [pH 7.2] and 2% osmium tetroxide), and the samples were washed 3 times with washing solution. Tissue dehydration was carried out through a series of acetone mixtures (25%, 50%, 75%, and 100% in ddH_2_O [v/v]) for 30 min each. The dehydrated samples were sequentially incubated in a gradient of Spurr resin (Sigma) solutions (25%, 50%, 75%, and 100% in acetone [v/v]) for 1 h each, and 100% Spurr overnight. For solidification, these samples were placed in moulds at 65°C for 2 days. Sections were taken with an Ultramicrotome (EM UC7, Leica), and observed with a transmission electron microscope (JEM‐2100F, JEOL).

### 4.8 Agronomic Traits of *OsFeSOD3* ‐Overexpressing Rice Under Drought Stress

To evaluate the agronomic traits of *OsFeSOD3*‐overexpressing transgenic rice under drought stress, 4‐month‐old wild‐type and *OsFeSOD3*‐overexpressing plants grown under field conditions at Kyungpook National University, Gunwi (128°34′ E, 36°15′ N), South Korea, were exposed to a 6‐day dehydration treatment. Following drought treatment, the plants were re‐irrigated and grown until harvest. At maturity, both wild‐type and transgenic rice were harvested and manually threshed. Growth and yield traits were assessed over two consecutive years (2023 and 2024), focusing on parameters such as plant height, number of tillers, heading date, number of spikelets, spikelets per tiller, number of grains, grain yield, grain filling rate, and 1000‐grain weight.

### 4.9 Yeast Two‐Hybrid Assay

To test the interaction between OsFeSOD3 and PEP complex components such as OspTAC10, OsFeSOD2, and OspTAC7, the yeast two‐hybrid assay was performed using the Matchmaker Gold Yeast Two‐Hybrid System (Clontech). Complementary DNAs (cDNAs) of *OsFeSOD3*, *OspTAC10*, *OsFeSOD2*, and *OspTAC7* were amplified by reverse transcription PCR from total RNA extracted from 1‐week‐old rice. The *OsFeSOD3* cDNA was introduced into the *Sma*I site of the bait vector pGBKT7, and the cDNAs of *OspTAC10*, *OsFeSOD2*, and *OspTAC7* were inserted into the *Sma*I site of the prey vector pGADT7 using the NEBuilder HiFi DNA Assembly System (New England Biolabs). The bait and prey constructs were co‐transformed into the Y2H Gold Yeast ( *Saccharomyces cerevisiae* ), and selected on minimal yeast growth medium without Trp and Leu (DDO). For protein–protein interaction analysis, selected yeast cells (OD_600_ = 0.05) were inoculated on a DDO medium containing 250 ng/mL aureobasidin A (Abs A) and incubated in the dark at 30°C for 3–4 days. Primer sequences used for vector construction are listed in Table S3.

### 4.10 GST Pull‐Down and co‐Immunoprecipitation Assays

GST pull‐down assays were performed as described by Zhang et al. (2005) with slight modifications (Zhang et al. 2005). *OsFeSOD3* cDNA was inserted into pGEX‐DC to express GST fusion proteins, and cDNAs of *OspTAC10*, *OsFeSOD2*, and *OspTAC7* were cloned into pMAL‐DC to express MBP fusion proteins. Recombinant proteins were expressed in *Escherichia coli* BL21 (DE3) pLysS cells by induction with 1 mM IPTG at 18°C overnight, and cells were lysed in homogenization buffer (25 mM Tris–HCl [pH 7.5], 0.5% Triton X‐100, 150 mM NaCl and 2 mM EDTA). For GST pull‐down assay, GST fusion proteins were immobilised on glutathione agarose beads (Thermo Fisher Scientific) at 4°C for 4 h with rotation, washed five times with the homogenization buffer without Triton X‐100, and then incubated with the MBP‐fused prey proteins at 4°C overnight. After five additional washes, proteins were eluted by boiling in 2× SDS sample buffer, separated on 8% SDS–PAGE, and transferred to PVDF membranes. Immunoblotting was performed using anti‐MBP antibody (Santa Cruz) and anti‐rabbit HRP‐linked secondary antibody (Abcam).

Co‐immunoprecipitation assays were performed as described by Yang et al. (2014) with slight modifications (Yang et al. 2014). Briefly, protoplasts were isolated from 1‐week‐old rice and co‐transformed with 35S::OsFeSOD3‐GFP and either 35S::OspTAC10‐Myc or 35S::OsFeSOD2‐Myc plasmids. The protoplasts were homogenised with immunoprecipitation (IP) buffer (25 mM Tris–HCl [pH 7.5], 150 mM NaCl, 0.5% Triton X‐100, 1 mM EDTA, 1 mM DTT and protease inhibitor cocktail) and centrifuged at 15000 × *g* for 15 min. The supernatants were incubated with anti‐GFP antibody (Thermo Fisher Scientific) overnight at 4°C, followed by addition of protein A/G agarose (Thermo Fisher Scientific) for 3 h at 4°C. Immunocomplexes were collected by centrifugation at 100 × *g* for 5 min, and washed four times with IP buffer and eluted by boiling in 2× sample buffer. Proteins were separated on 8% SDS–PAGE, transferred to PVDF membranes, immunoblotted with anti‐Myc antibody (Abcam). Immunoblot signals were detected with West‐Q Pico ECL Solution (GeneDEPOT) and visualised using the iBright CL1500 Imaging System (Thermo Fisher Scientific).

### 4.11 Statistical Analyses

Data are presented as mean values, and the number of samples is indicated in the figure legends. The statistical difference between the samples and their controls was determined using a 2‐tailed *t*‐test with a *p* < 0.01.

### 4.12 Accession Numbers

Sequence data from this article can be found in the GenBank/EMBL data libraries under the following accession numbers: *Arabidopsis FeSOD3*; AT5G23310, *OsFeSOD3*; NP_001389670, Barley *FeSOD3*; XP_044961365, Maize *FeSOD3*; NP_001104871, Wheat *FeSOD3*; XP_044366067, Tobacco *FeSOD3*; XP_016501759, Tomato *FeSOD3*; NP_001300698, Moss *FeSOD3*; XP_024379755, *OsRbcL*; YP_654221, *OsTUB2*; NP_001391388, *OsActin*; NP_001389172, *OsPsbA*; NP_039360, *OsPsbB*; BAD32930, *OsPetD*; NP_039416; *OspTAC2*; XP_015631414, *OspTAC3*; NP_001368793, *OspTAC6*; XP_015617226, *OspTAC7*; XP_015621671, *OspTAC10*; XP_015616255, *OspTAC12*; XP_015621331, *OspTAC14*; XP_015637857, *OspTAC18*; QGX02124, *OsFeSOD2*; WWB08873, *OsFLN1*; XP_015616764, *OsFLN2*; XP_025879439, *OsMurE‐like*; XP_015614047, *OsPRIN2*; NP_001409679, *OsTrxZ*; NP_001409476.

## Author Contributions

G.J. conceived the original screening and research plans. G.J. designed and supervised the experiments. D.H.S. and J.J. performed the experiments and analysed the data. G.J. wrote the article with contributions from all authors.

## Funding

This work was supported by the National Research Foundation of Korea grant funded by the Ministry of Science and ICT (RS‐2025‐00519942), and Global—Learning & Academic research institution for Master's·PhD students and Postdocs (LAMP) Program of the National Research Foundation of Korea grant funded by the Ministry of Education (RS‐2024‐00442775). This work was also supported by the New Breeding Technologies Development Program (RS‐2024‐00322111), Rural Development Administration, Korea.

## Conflicts of Interest

The authors declare no conflicts of interest.

## Supporting information


**Data S1:** Supporting Information.
**Figure S1:** Salt stress‐induced chloroplastic ROS accumulation.
**Figure S2:** Chloroplast‐specific ROS accumulation under oxidative stress conditions.
**Figure S3:** Identification of OsFeSOD3.
**Figure S4:** Molecular characterisation of *OsFeSOD3*‐overexpressing rice.
**Figure S5:**. Growth of *OsFeSOD3*‐overexpressing rice at vegetative stage.
**Figure S6:**. Regulation of chloroplastic ROS accumulation by *OsFeSOD3*.
**Figure S7:** pbi70508‐sup‐0001‐DataS1.pdf. *OsFeSOD3* overexpression reduces H_2_O_2_ levels.
**Figure S8:**. Suppression of chloroplastic ROS accumulation by *OsFeSOD3* overexpression under drought stress.
**Figure S9:**. Drought tolerance improvement by *OsFeSOD3* overexpression.
**Figure S10:** pbi70508‐sup‐0001‐DataS1.pdf. *OsFeSOD3* mutants exhibit an albino phenotype.
**Figure S11:** Structural modelling of OsFeSOD3 interactions with rice PEP complex components.
**Figure S12:** Interaction of OsFeSOD3 with rice PEP complex components.
**Figure S13:** Expression patterns of *OsFeSOD3*.
**Figure S14:** Working model for the bifunctional role of OsFeSOD3.
**Figure S15:** *OsFeSOD3* overexpression delays the onset of cytoplasmic ROS accumulation.
**Figure S16:** Enhanced salt tolerance conferred by *OsFeSOD3* overexpression.
**Figure S17:** *OsFeSOD3* overexpression improves oxidative stress tolerance.
**Table S1:** Agronomic traits of *OsFeSOD3*‐overexpressing rice.
**Table S2:** AlphaFold‐Multimer prediction showing possible interactions of OsFeSOD3 with rice PEP‐complex components.
**Table S3:** Primers used in this study.

## Data Availability

All data in this study are included in this published article and its Supporting Information S1.
