# Supplementary material for: OsFeSOD3 Functions as an Enzymatic Component of the PEP Complex, Bifunctionally Regulating Chloroplastic ROS Metabolism and Chloroplast Biogenesis in Rice
Source: Plant Biotechnol J. 2025 Dec 17;24(4):2475–91. doi: 10.1111/pbi.70508 (PMC13140286; doi:10.1111/pbi.70508)
Supplement: Supplementary file 1 — Data S1: Supporting Information. Figure S1: Salt stress‐induced chloroplastic ROS accumulation. Figure S2: Chloroplast‐specific ROS accumulation under oxidative stress conditions. Figure S3: Identification of OsFeSOD3. Figure S4: Molecular characterisation of OsFeSOD3‐overexpressing rice. Figure S5:. Growth of OsFeSOD3‐overexpressing rice at vegetative stage. Figure S6:. Regulation of chloroplastic ROS accumulation by OsFeSOD3. Figure S7: pbi70508‐sup‐0001‐DataS1.pdf. OsFeSOD3 overexpression reduces H2O2 levels. Figure S8:. Suppression of chloroplastic ROS accumulation by OsFeSOD3 overexpression under drought stress. Figure S9:. Drought tolerance improvement by OsFeSOD3 overexpression. Figure S10: pbi70508‐sup‐0001‐DataS1.pdf. OsFeSOD3 mutants exhibit an albino phenotype. Figure S11: Structural modelling of OsFeSOD3 interactions with rice PEP complex components. Figure S12: Interaction of OsFeSOD3 with rice PEP complex components. Figure S13: Expression patterns of OsFeSOD3. Figure S14: Working model for the bifunctional role of OsFeSOD3. Figure S15: OsFeSOD3 overexpression delays the onset of cytoplasmic ROS accumulation. Figure S16: Enhanced salt tolerance conferred by OsFeSOD3 overexpression. Figure S17: OsFeSOD3 overexpression improves oxidative stress tolerance. Table S1: Agronomic traits of OsFeSOD3‐overexpressing rice. Table S2: AlphaFold‐Multimer prediction showing possible interactions of OsFeSOD3 with rice PEP‐complex components. Table S3: Primers used in this study. [file PBI-24-2475-s001.pdf]

Supporting Information

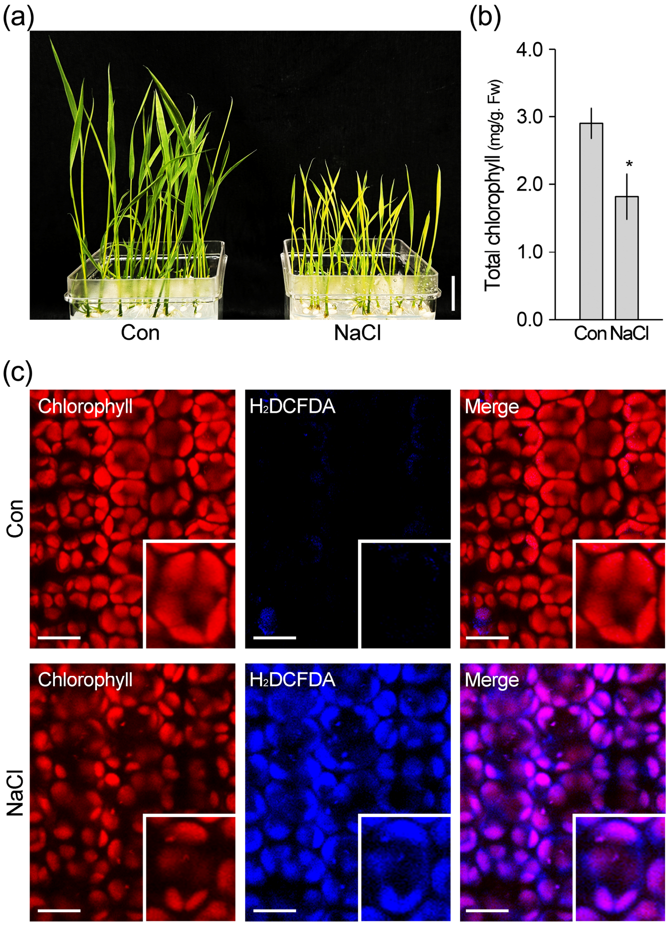

Supplemental Figure S1. Salt stress-induced chloroplastic ROS accumulation.

(a) Images of 2-week-old wild-type rice grown in normal ½ MS medium (Con) or ½ MS medium containing 150 mM NaCl (NaCl). (b) Quantification of total chlorophyll content in these plants. (c) Visualization of chloroplastic ROS accumulation under salt stress using H_2DCFDA staining. Red and blue fluorescence correspond to chlorophyll auto-fluorescence and H_2DCFDA signals, respectively. Quantification data are the means of seven biological replicates. Error bars indicate SD. The asterisk indicates statistically significant differences between the samples and controls ($P < 0.01$, two-tailed *t*-test). Scale bars = 2 cm in (a) and 5 μm in (c).

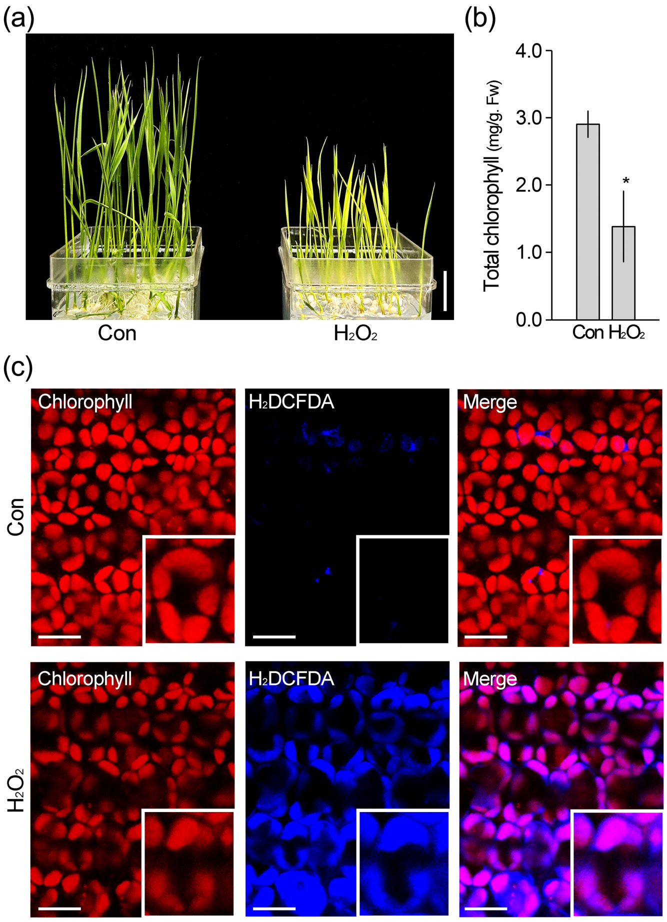

Supplemental Figure S2. Chloroplast-specific ROS accumulation under oxidative stress conditions.

(a) Morphology of wild-type rice grown in normal ½ MS medium (Con) or ½ MS medium containing 10 mM H_2O_2 (H_2O_2) for 2 weeks. (b) A graph showing the total chlorophyll content in these plants. (c) H_2DCFDA staining showing ROS accumulation in chloroplasts under oxidative conditions. Red and blue fluorescence correspond to chlorophyll auto-fluorescence and H_2DCFDA signals, respectively. Quantification data are the means of seven biological replicates. Error bars indicate SD. The asterisk indicates statistically significant differences between the samples and controls ($P < 0.01$, two-tailed *t*-test). Scale bars = 2 cm in (a) and 5 μm in (c).

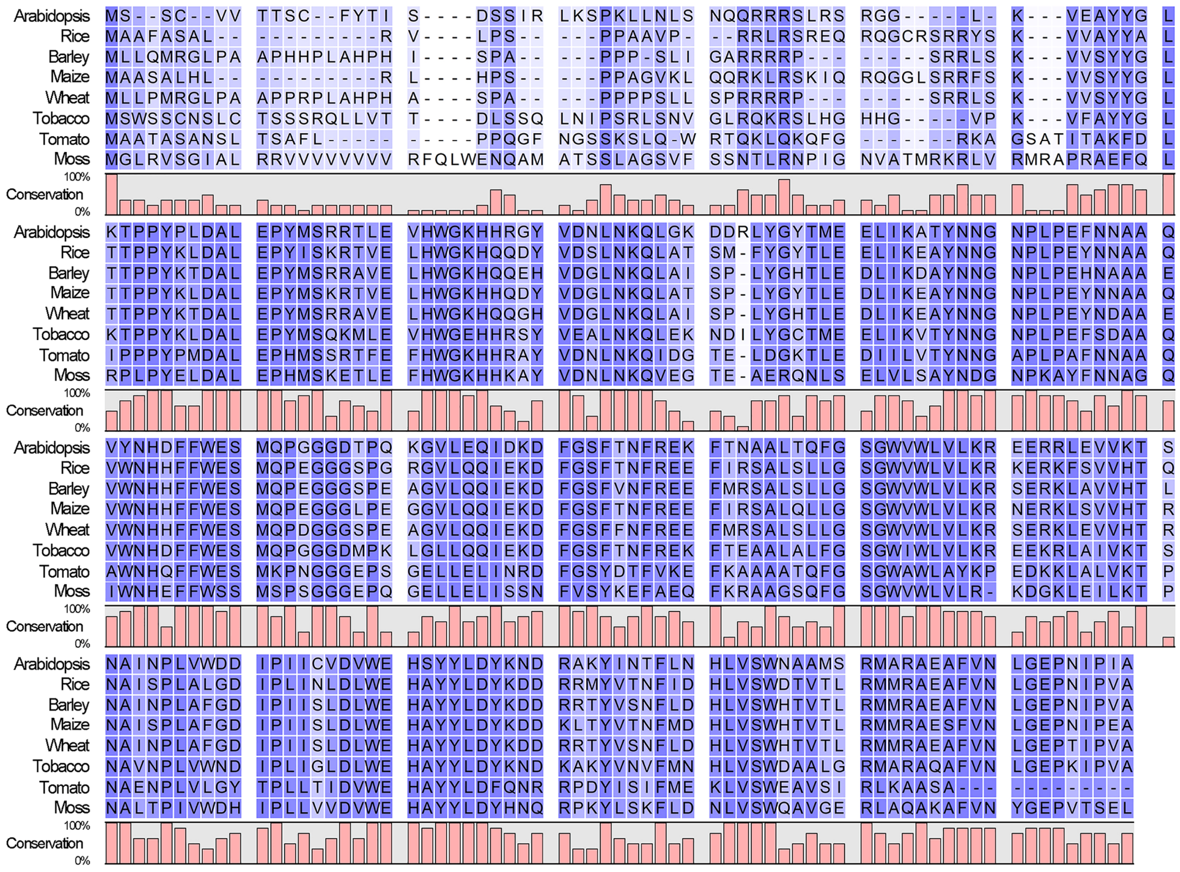

Supplemental Figure S3. Identification of OsFeSOD3.

Amino acid sequence alignment of the FeSOD3 protein homologs in *Arabidopsis thaliana*, rice (*Oryza sativa*), barley (*Hordeum vulgare*), maize (*Zea mays*), wheat (*Triticum aestivum*), tobacco (*Nicotiana tabacum*), tomato (*Solanum lycopersicum*) and moss (*Physcomitrella patens*). The graph indicates the degree of their conservation, with darker blue boxes indicating highly conserved amino acid sequences.

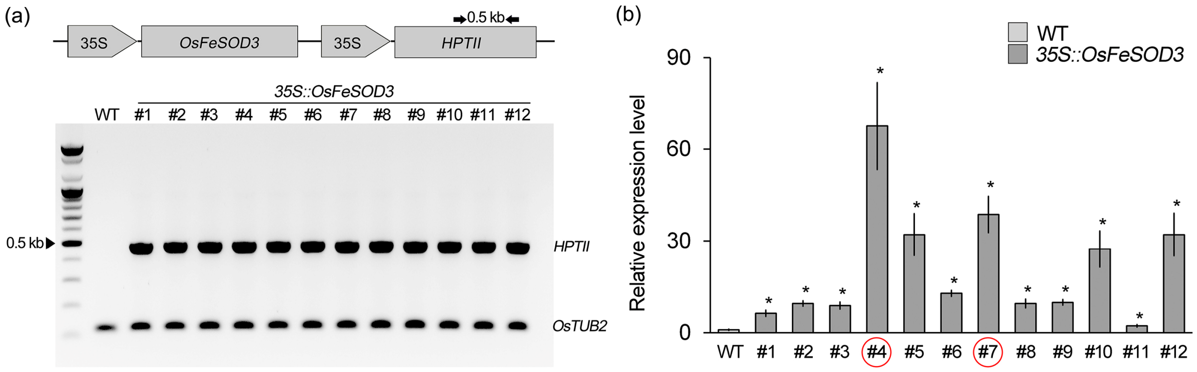

Supplemental Figure S4. Molecular characterization of *OsFeSOD3*-overexpressing rice. (a) A schematic of the recombinant plasmid structure for *OsFeSOD3*-overexpression (top) and PCR amplification results (bottom) showing that 12 independent lines of T_0 *OsFeSOD3*-overexpressing plants (*35S::OsFeSOD3*) carry the transgene, unlike wild-type plants (WT). *OsTUB2* was used as a control for PCR amplification. Black arrows indicate the positions where primers were designed for the amplification. (b) Analysis of *OsFeSOD3* expression levels in the 12 independent lines of T_0 *OsFeSOD3*-overexpressing plants. The line #4 and 7, marked by red circles, were selected for further experiments. *OsTUB2* was used as a reference gene to normalize the RT-qPCR results. RT-qPCR data are the means of 3 technical replicates, and error bars indicate SD. Asterisks indicate statistically significant differences between the corresponding samples and their controls ($P < 0.01$, two-tailed t-test).

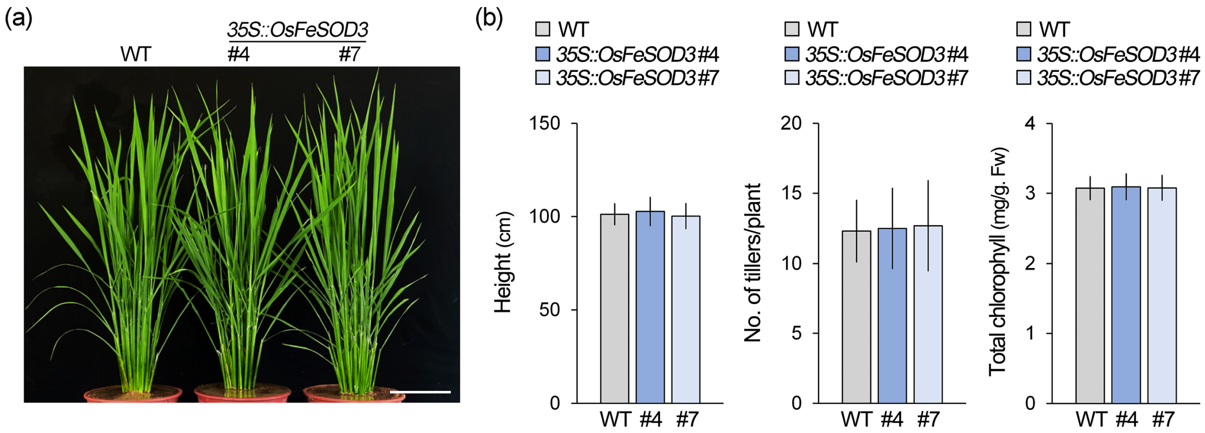

Supplemental Figure S5. Growth of *OsFeSOD3*-overexpressing rice at vegetative stage. (a) Image of wild-type (WT) and *OsFeSOD3*-overexpressing plants (*35S::OsFeSOD3*) grown in soils for 3 months. #4 and 7 indicate two independent T_3 lines of *35S::OsFeSOD3* plants. (b) Quantification of plant height (left), the number of tillers per plant (middle), and total chlorophyll content (right) in these plants (n > 64). Quantification data are the means of six biological replicates. Error bars indicate SD. Scale bar = 20 cm.

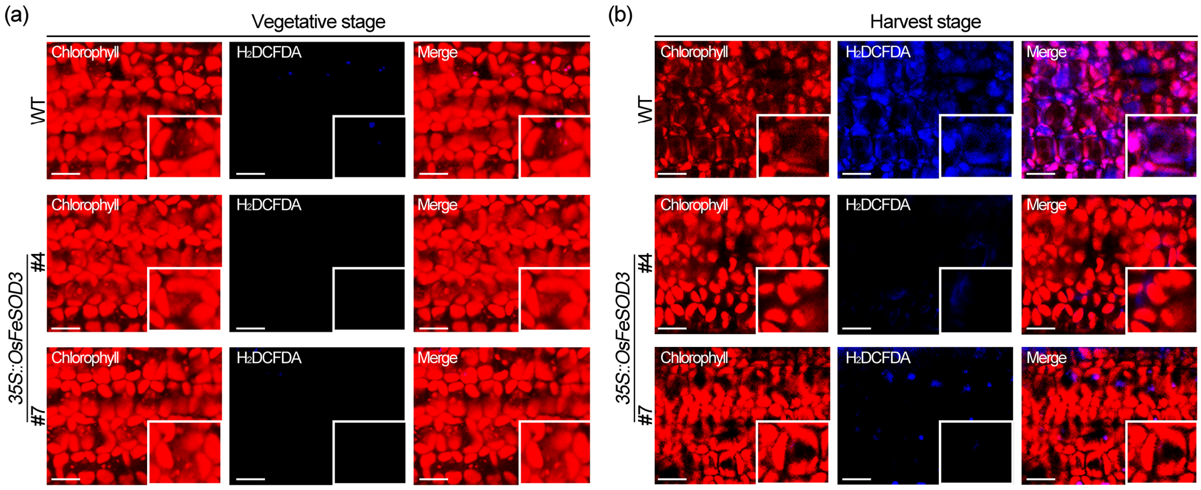

Supplemental Figure S6. Regulation of chloroplastic ROS accumulation by *OsFeSOD3*. (a, b) H_2DCFDA staining results showing chloroplastic ROS accumulation in leaves of wild-type and *OsFeSOD3*-overexpressing rice. (a) Leaves collected from 3-month-old plants at the vegetative stage. (b) Leaves collected from 5-month-old plants at the harvest stage. #4 and 7 indicate two independent T_3 lines of *35S::OsFeSOD3* rice. Red and blue fluorescence correspond to chlorophyll autofluorescence and H_2DCFDA signals, respectively. Scale bars = 5 μm.

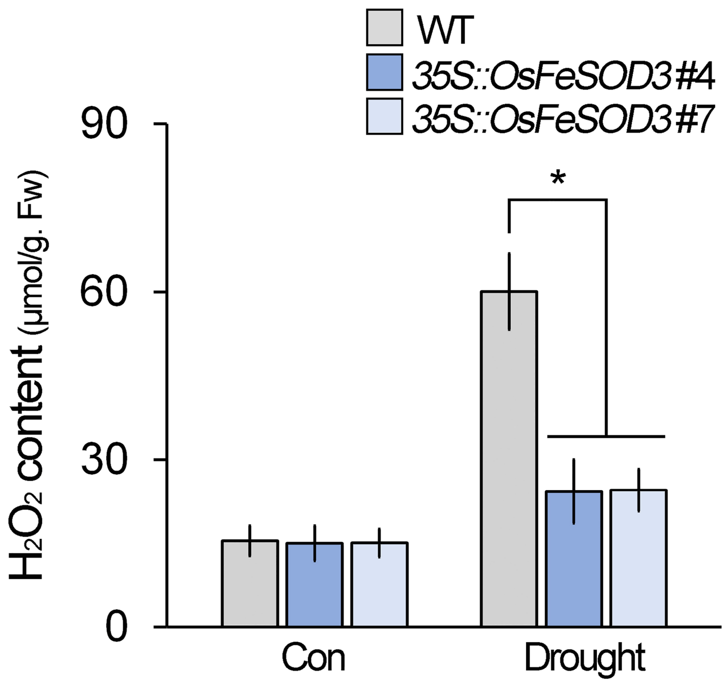

Supplemental Figure S7. *OsFeSOD3* overexpression reduces H_2O_2 levels.

Quantification of H_2O_2 content in 4-week-old wild-type (WT) and *OsFeSOD3*-overexpressing plants (*35S::OsFeSOD3*) under normal conditions (Con) and after 2 days of drought stress (Drought). #4 and 7 indicate two independent T_4 lines of *35S::OsFeSOD3* rice. Data represent the means of seven biological replicates. The asterisk indicates statistically significant differences between the corresponding samples and their controls ($P < 0.01$, two-tailed *t*-test).

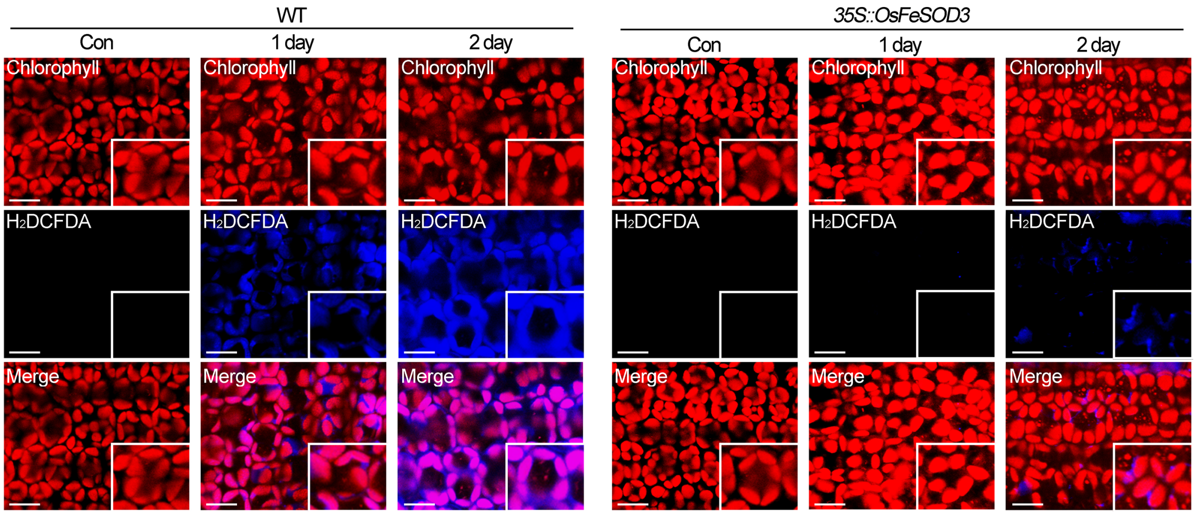

Supplemental Figure S8. Suppression of chloroplastic ROS accumulation by *OsFeSOD3* overexpression under drought stress.

H_2DCFDA staining results showing chloroplastic ROS accumulation in 4-week-old wild-type (WT) and T_4 *OsFeSOD3*-overexpressing plants (*35S::OsFeSOD3*) after 1 and 2 days of drought stress. Red and blue fluorescence correspond to chlorophyll autofluorescence and H_2DCFDA signals, respectively. Scale bars = 5 μm.

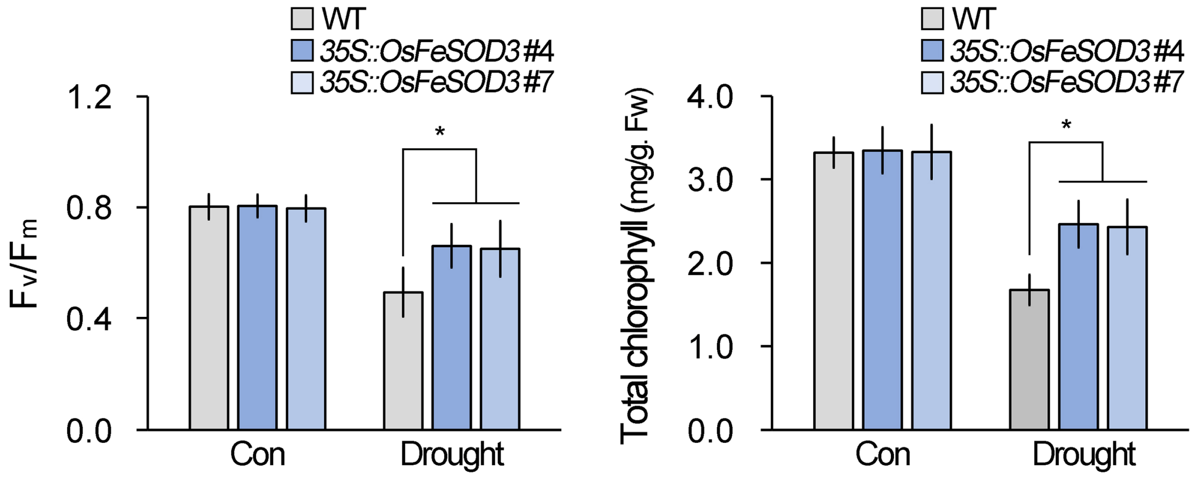

Supplemental Figure S9. Drought tolerance improvement by *OsFeSOD3* overexpression. Changes in photosynthetic efficiency (left) and chlorophyll content (right) in wild-type and *OsFeSOD3*-overexpressing plants at the reproductive stage. Photosynthetic efficiency and chlorophyll content were measured in 4-month-old wild-type and *35S::OsFeSOD3* plants after 2 days of drought stress treatment (n > 42). #4 and 7 indicate two independent T_3 lines of *35S::OsFeSOD3* plants. Quantification data are the means of three biological replicates. Error bars indicate SD. Asterisks indicate statistically significant differences between the samples and their controls ($P < 0.01$, two-tailed t-test).

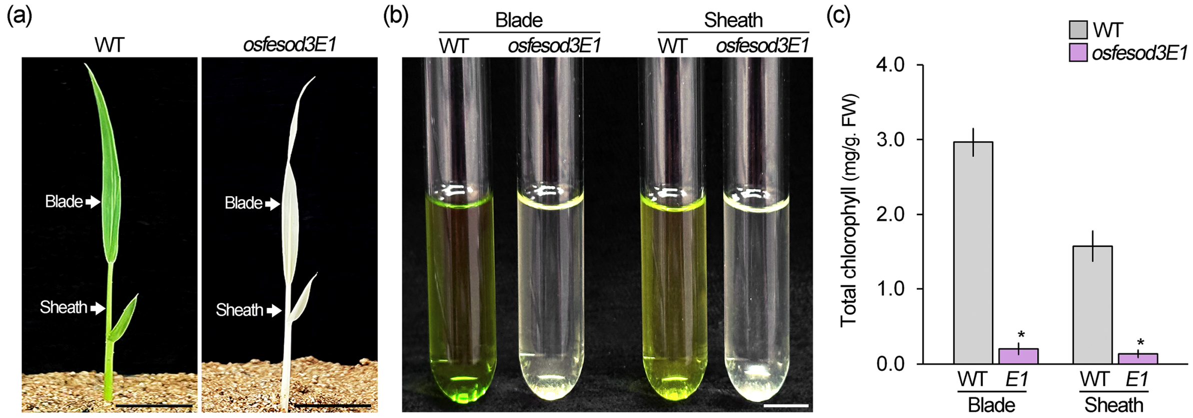

Supplemental Figure S10. *OsFeSOD3* mutants exhibit an albino phenotype.

(a) Images of 1-week-old wild-type (WT) and *OsFeSOD3* knock-out mutant (*osfesod3E1*) plants. (b) Chlorophyll extracts prepared from the leaf blades (Blade) and leaf sheaths (Sheath) of the indicated plants. (c) Quantification of total chlorophyll content in these samples. *E1* indicates the *osfesod3E1* mutant plants. Quantification data are the means of seven biological replicates. Error bars indicate SD. Asterisks indicate statistically significant differences between the corresponding samples and their controls ($P < 0.01$, two-tailed t-test). Scale bars = 1 cm.

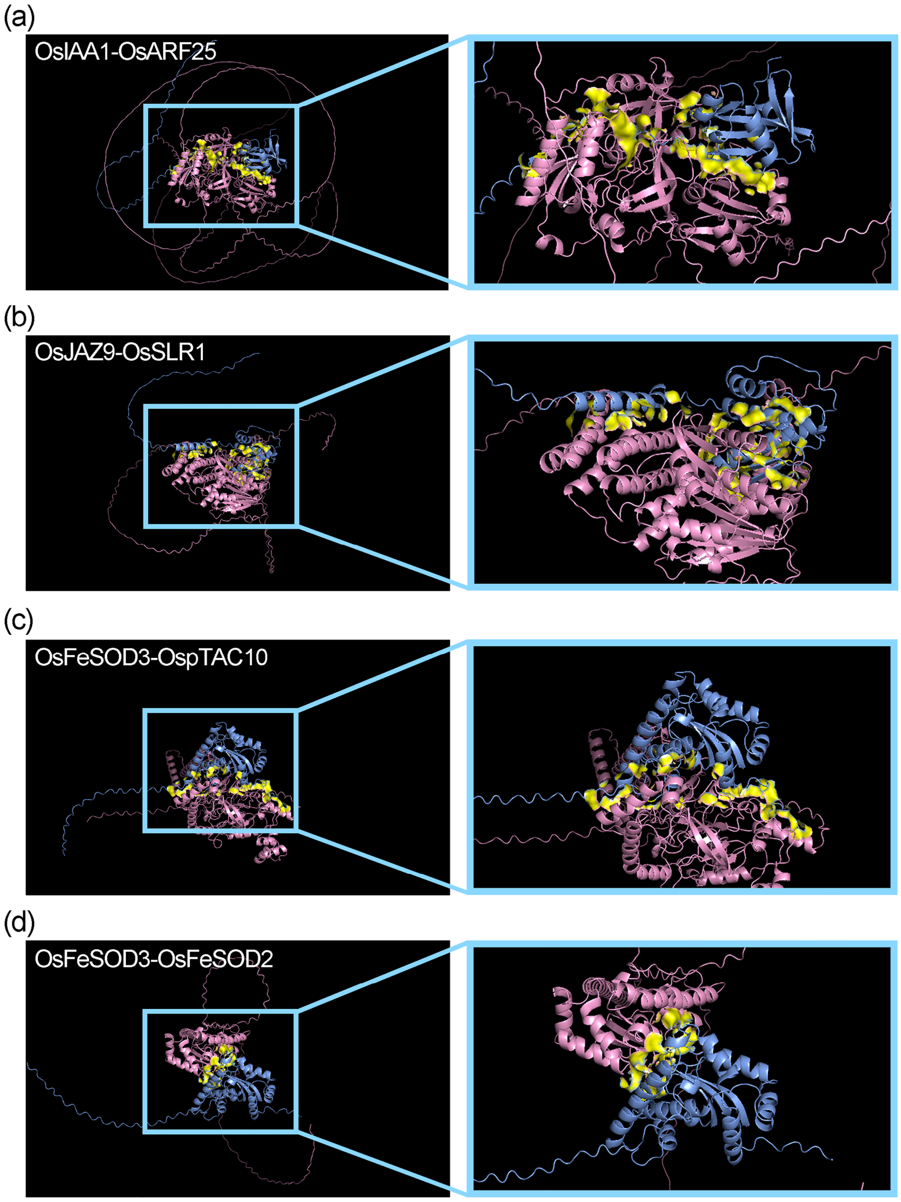

Supplemental Figure S11. Structural modeling of OsFeSOD3 interactions with rice PEP complex components.

AlphaFold-Multimer-based structural models showing interactions of OsIAA1–OsARF25 (a), OsJAZ9–OsSLR1 (b), OsFeSOD3–OspTAC10 (c), and OsFeSOD3–OsFeSOD2 (d). Left panels show the overall structures, and right panels show high-magnification views of the interaction interfaces (Abramson et al., 2024; https://alphafoldserver.com/welcome). OsIAA1, OsJAZ9, and OsFeSOD3 are colored in blue, and their interacting partners (OsARF25, OsSLR1, OspTAC10, and OsFeSOD2) are shown in pink. Residues within 4 Å between the indicated proteins are highlighted in yellow, suggesting potential involvement in the predicted interactions (Li et al., 2022; Um et al., 2018; Wu et al., 2024a).

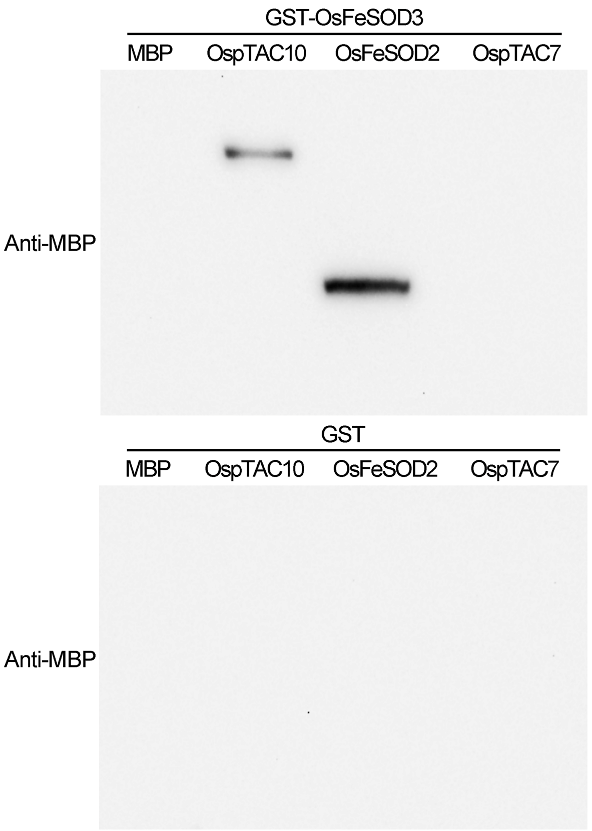

Supplemental Figure S12. Interaction of OsFeSOD3 with rice PEP complex components. GST pull-down assay showing interactions of OsFeSOD3 with rice PEP complex components (OspTAC10, OsFeSOD2, and OspTAC7). GST-OsFeSOD3 proteins were immobilized on glutathione agarose beads and incubated with MBP-OspTAC10 (OspTAC10), MBP-OsFeSOD2 (OsFeSOD2) and MBP-OspTAC7 (OspTAC7). Immunoblotting was performed with an anti-MBP antibody, and MBP and GST proteins were used as negative controls.

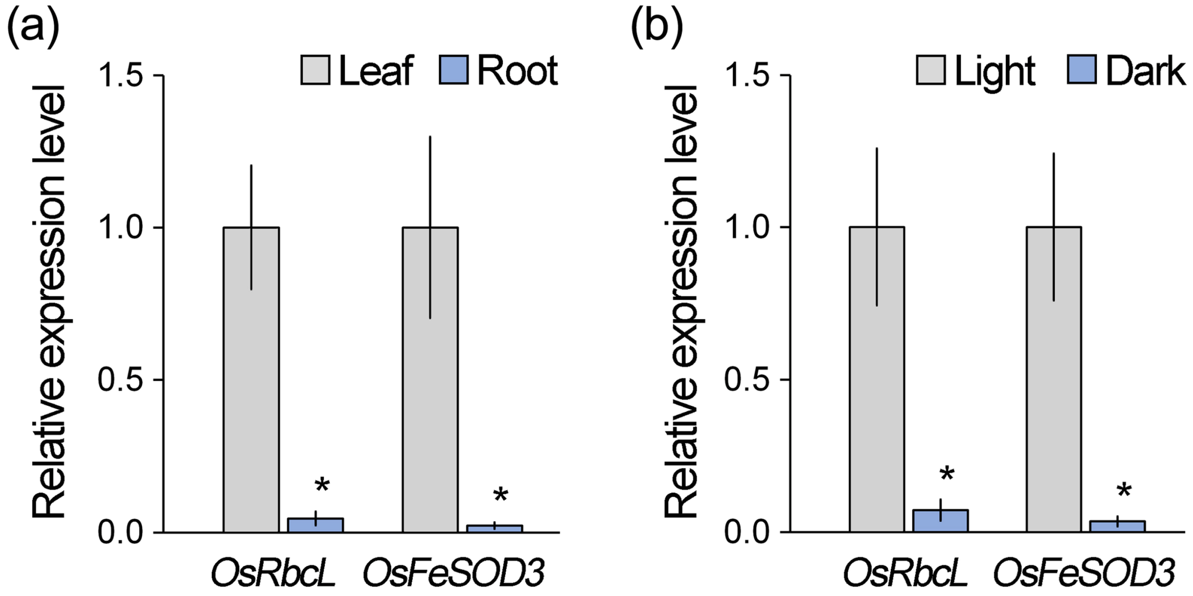

Supplemental Figure S13. Expression patterns of *OsFeSOD3*.

(a, b) RT-qPCR results showing the leaf-specific (a) and light-dependent (b) expression patterns of *OsFeSOD3*. For the analysis of tissue-specific expression, total RNA was extracted from WT leaves (Leaf) and roots (Root) grown in ½ MS medium under a light regime of 16 h/8 h light/dark for 1 week. For the analysis of light-dependent expression, total RNA was extracted from wild-type (WT) rice grown in ½ MS medium under continuous light (Light) or dark (Dark) conditions for 1 week. *OsTUB2* was used as a reference gene to normalize the RT-qPCR results. Data are the means of 9 replicates (3 biological × 3 technical). Error bars indicate SD. Asterisks indicate statistically significant differences between the corresponding samples and their controls ($P < 0.01$, two-tailed *t*-test).

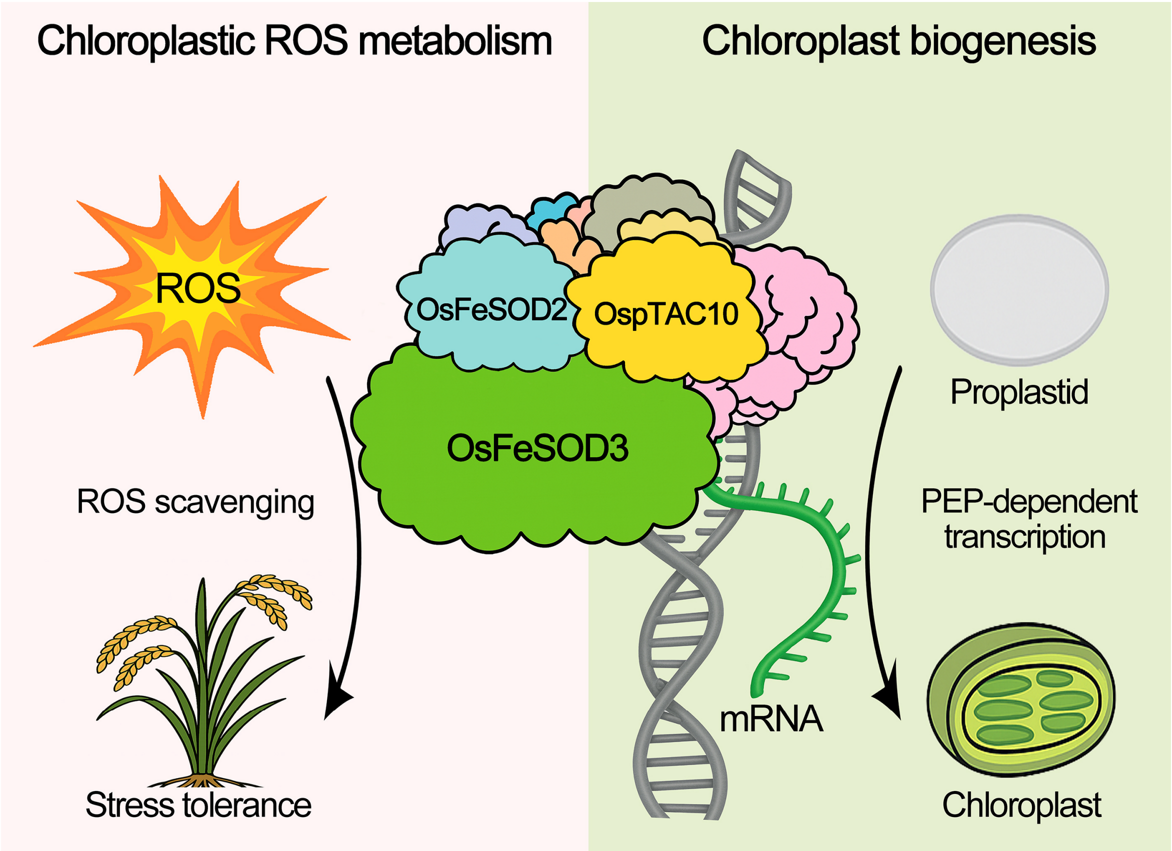

Supplemental Figure S14. Working model for the bifunctional role of OsFeSOD3.
A schematic model showing OsFeSOD3 as an enzymatic component of the PEP complex, bifunctionally regulating chloroplastic ROS metabolism and chloroplast biogenesis in rice.

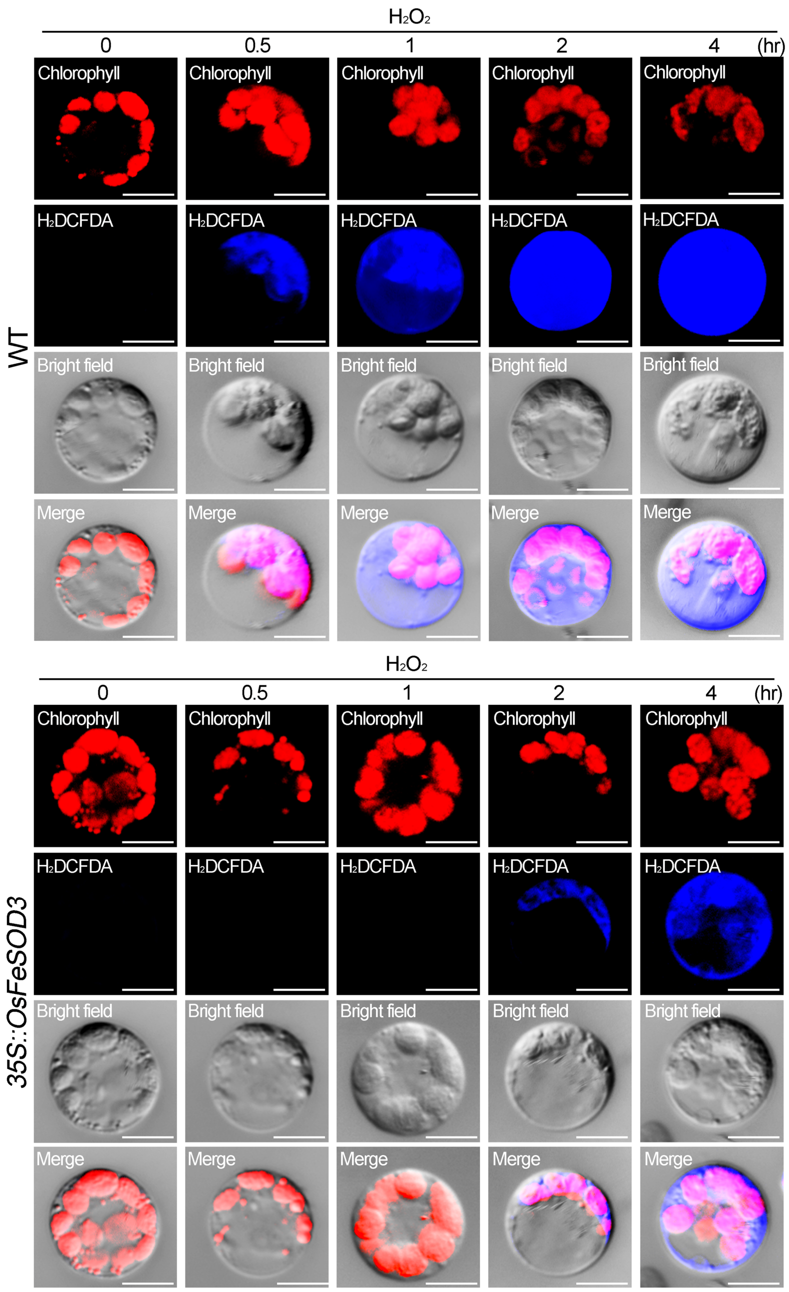

Supplemental Figure S15. *OsFeSOD3* overexpression delays the onset of cytoplasmic ROS accumulation.

Time-course monitoring of H_2DCFDA fluorescence signals in wild-type and *35S::OsFeSOD3* protoplasts treated with 2 mM H_2O_2 for 0.5, 1, 2 and 4 hours. Red and blue fluorescence correspond to chlorophyll autofluorescence and H_2DCFDA signals. Scale bars = 10 μm.

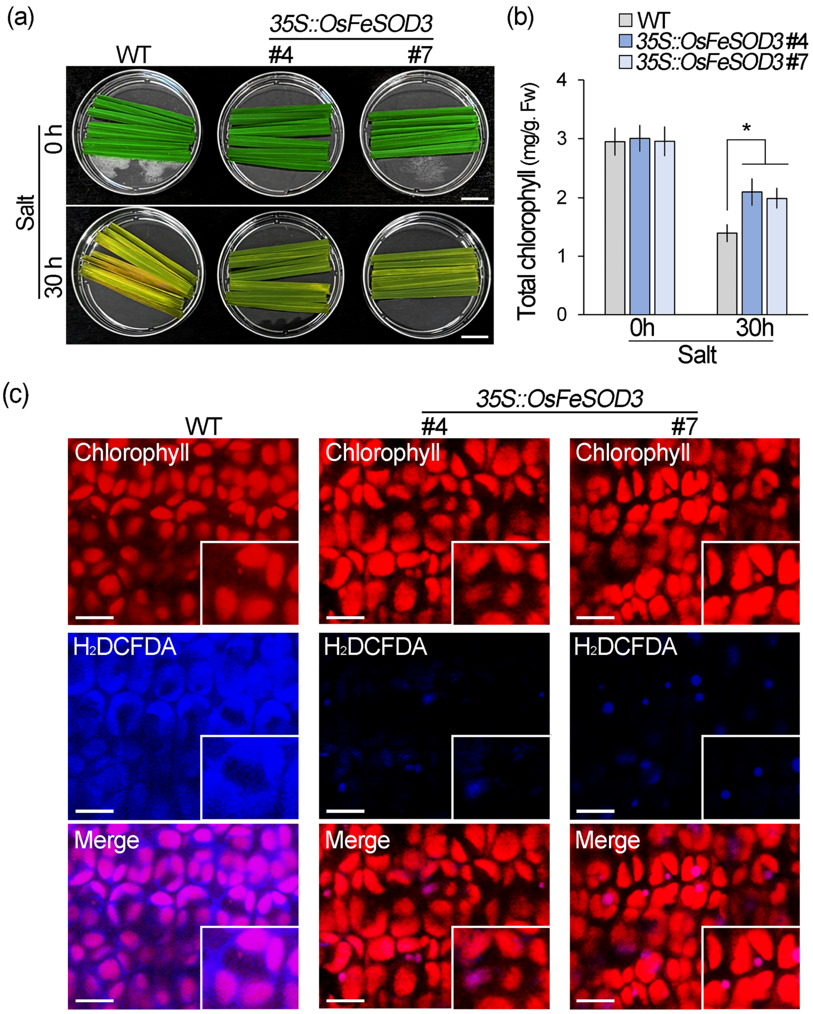

Supplemental Figure S16. Enhanced salt tolerance conferred by *OsFeSOD3* overexpression.

(a) Leaf phenotypes of wild-type (WT) and *OsFeSOD3*-overexpressing (*35S::OsFeSOD3*) plants under salt stress. (b) Quantification of total chlorophyll content in these plants. #4 and 7 represent two independent T_4 lines of *35S::OsFeSOD3*. Leaves were collected from 4-week-old WT and *35S::OsFeSOD3* plants and incubated in 400 mM NaCl for 30 h. (c) Visualization of ROS accumulation in chloroplasts using H_2DCFDA in these plants. Quantification data are the means of seven biological replicates. Error bars indicate SD. The asterisk indicates statistically significant differences between the corresponding samples and their controls (P < 0.01, two-tailed t-test). Scale bars = 2 cm in (a) and 5 μm in (c).

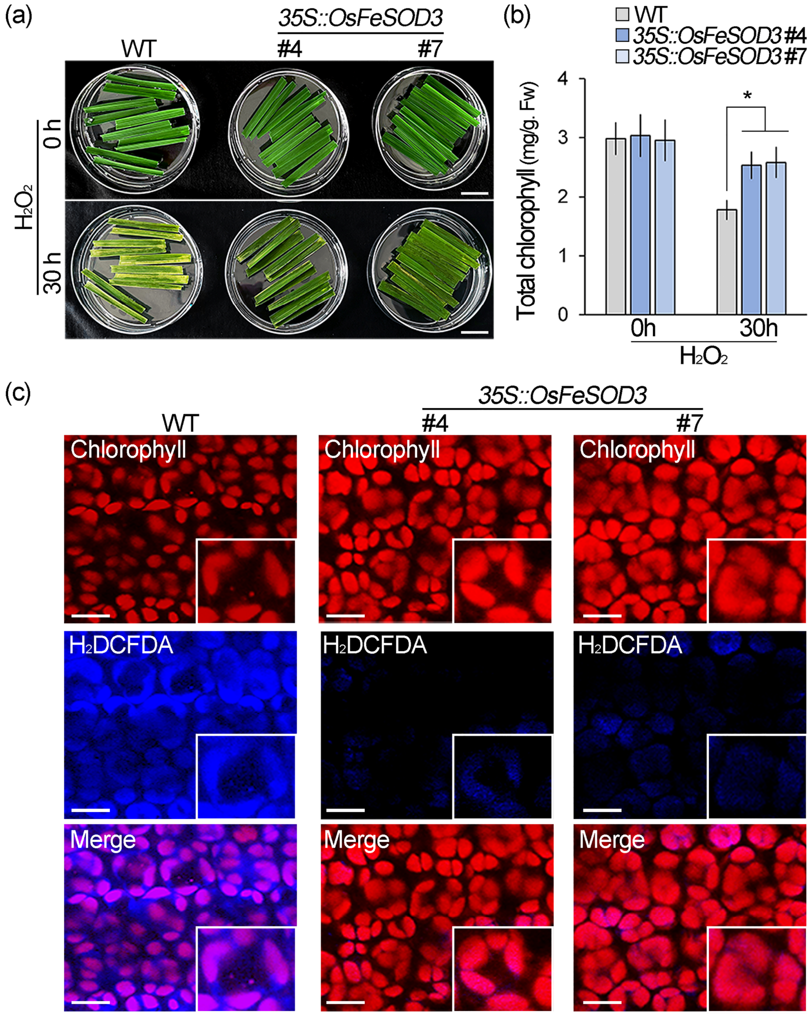

Supplemental Figure S17. *OsFeSOD3* overexpression improves oxidative stress tolerance. (a) Leaf disc assay of wild-type (WT) and *OsFeSOD3*-overexpressing (*35S::OsFeSOD3*) plants under H_2O_2 treatment. (b) Quantification of total chlorophyll content in these plants. #4 and 7 indicate two independent T_4 lines of *35S::OsFeSOD3* plants. Leaves were collected from 4-week-old wild-type and *35S::OsFeSOD3* plants, and incubated in 500 mM H_2O_2 for 30 h. (c) Visualization of chloroplastic ROS accumulation using H_2DCFDA staining in these plants. Quantification data are the means of seven biological replicates. Error bars indicate SD. The asterisk indicates statistically significant differences between the corresponding samples and their controls ($P < 0.01$, two-tailed *t*-test). Scale bars = 2 cm in (a) and 5 μm in (c).

Table S1. Agronomic traits of *OsFeSOD3*-overexpressing rice.

[Normal conditions]

	Height (cm)	No. of tillers /plant	Heading date (days after sowing; DAS)	No. of spikelets /plant	No. of spikelets /tiller	Grain filling rate (%)	No. of grains /plant	1000-grain weight /plant (g)	Grain yield /plant (g)
**2023**									
WT	105.38 ± 4.05	12.85 ± 1.52	108.04 ± 1.35	1291.72 ± 187.68	100.58 ± 9.53	88.91 ± 5.78	1146.73 ± 173.38	20.14 ± 1.37	22.99 ± 2.98
35S::OsFeSOD3 #4	106.09 ± 3.03	12.80 ± 1.69	108.09 ± 1.37	1296.07 ± 226.25	100.94 ± 9.37	90.89 ± 4.83	1179.41 ± 224.10	19.99 ± 1.76	23.34 ± 3.62
%Δ	+ 0.67	- 0.37	+ 0.04	+ 0.33	+ 0.35	+ 2.23	+ 2.85	- 0.73	+ 1.54
35S::OsFeSOD3 #7	106.42 ± 6.41	12.61 ± 1.20	107.95 ± 1.77	1324.28 ± 176.88	104.84 ± 7.87	90.40 ± 4.33	1194.52 ± 146.74	19.67 ± 1.34	23.33 ± 1.66
%Δ	+ 0.99	- 1.85	- 0.08	+ 2.52	+ 4.24	+ 1.68	+ 4.16	- 2.34	+ 1.50
**2024**									
WT	104.04 ± 5.40	13.00 ± 1.78	106.57 ± 1.02	1329.88 ± 216.23	102.08 ± 5.60	90.72 ± 6.48	1199.41 ± 168.18	20.28 ± 1.77	24.23 ± 3.23
35S::OsFeSOD3 #4	104.66 ± 4.58	12.80 ± 1.60	106.66 ± 1.35	1314.49 ± 195.83	102.55 ± 7.07	93.16 ± 3.35	1229.01 ± 215.78	20.40 ± 1.47	24.85 ± 3.15
%Δ	+ 0.59	- 1.46	+ 0.08	- 1.15	+ 0.46	+ 2.69	+ 2.46	+ 0.58	+ 2.56
35S::OsFeSOD3 #7	103.57 ± 4.33	12.90 ± 1.78	106.76 ± 1.26	1342.77 ± 182.92	104.28 ± 6.11	90.99 ± 4.08	1218.73 ± 150.10	20.48 ± 1.05	24.92 ± 2.88
%Δ	- 0.45	- 0.73	+ 0.17	+ 0.96	+ 2.15	+ 0.30	+ 1.61	+ 0.94	+ 2.84

[Drought conditions]

	Height (cm)	No. of tillers /plant	Heading date (days after sowing; DAS)	No. of spikelets /plant	No. of spikelets /tiller	Grain filling rate (%)	No. of grains /plant	1000-grain weight /plant (g)	Grain yield /plant (g)
**2023**									
WT	105.09 ± 3.78	12.52 ± 1.66	108.09 ± 1.09	1274.21 ± 259.09	101.46 ± 14.08	43.83 ± 8.97	545.40 ± 102.70	17.50 ± 2.71	9.41 ± 1.63
35S::OsFeSOD3 #4	105.90 ± 3.64	12.66 ± 1.62	108.00 ± 1.14	1271.16 ± 154.44	100.73 ± 8.19	60.87 ± 7.48 *	769.25 ± 101.37 *	17.54 ± 2.13	13.37 ± 1.59 *
%Δ	+ 0.77	+ 1.14	- 0.08	- 0.23	- 0.72	+ 38.86	+ 41.04	+ 0.21	+ 42.15
35S::OsFeSOD3 #7	106.23 ± 4.42	12.61 ± 1.20	108.00 ± 1.58	1305.67 ± 244.56	103.34 ± 15.18	57.94 ± 8.76 *	762.93 ± 227.25 *	17.91 ± 2.60	13.17 ± 1.82 *
%Δ	+ 1.08	+ 0.76	- 0.08	+ 2.46	+ 1.85	+ 32.19	+ 39.88	+ 2.31	+ 40.03
**2024**									
WT	103.95 ± 3.99	12.89 ± 1.37	106.52 ± 1.07	1294.48 ± 164.38	103.67 ± 11.79	42.77 ± 9.09	554.01 ± 133.67	17.36 ± 2.11	9.59 ± 2.45
35S::OsFeSOD3 #4	104.81 ± 4.46	12.66 ± 1.90	106.76 ± 1.17	1293.80 ± 258.34	101.62 ± 9.94	62.69 ± 8.31 *	796.18 ± 113.79 *	17.17 ± 1.98	13.52 ± 1.41 *
%Δ	+ 0.82	- 1.76	+ 0.22	- 0.05	- 1.97	+ 46.55	+ 43.71	- 1.07	+ 40.90
35S::OsFeSOD3 #7	104.19 ± 4.33	12.71 ± 1.70	106.57 ± 0.74	1283.42 ± 147.13	101.32 ± 5.43	57.00 ± 6.90 *	728.66 ± 103.47 *	17.54 ± 1.66	12.71 ± 1.66 *
%Δ	+ 0.22	- 1.39	+ 0.04	- 0.85	- 2.26	+ 33.25	+ 31.52	+ 1.05	+ 32.53

Growth and productivity data under normal and drought conditions were collected across two seasons, 2023 (T_3) and 2024 (T_4) ($n > 21$ for each year). The %Δ indicates the percentage difference between wild type (WT) and *OsFeSOD3*-overexpressing plants (*35S::OsFeSOD3*). #4 and 7 indicate two independent lines of *35S::OsFeSOD3* plants. Asterisks indicate statistically significant differences between WT and *35S::OsFeSOD3* plants ($P < 0.01$, two-tailed *t*-test).

Table S2. AlphaFold-Multimer prediction showing possible interactions of OsFeSOD3 with rice PEP-complex components.

Protein 1	Protein 2	ipTM	pTM	AFM model confidence score
OsIAA1	OsARF25	0.540	0.410	0.514
OsJAZ9	OsSLR1	0.520	0.560	0.528
OsFeSOD3	OsFeSOD2	0.780	0.680	0.760
	OspTAC10	0.670	0.550	0.646
	OspTAC14	0.340	0.630	0.398
	OsFLN2	0.320	0.490	0.354
	OspTAC3	0.200	0.550	0.270
	OspTAC6	0.230	0.400	0.264
	OsTrxZ	0.190	0.510	0.254
	OsMurE-like	0.160	0.540	0.236
	OspTAC12	0.200	0.330	0.226
	OspTAC2	0.150	0.520	0.224
	OspTAC18	0.130	0.520	0.208
	OsPRIN2	0.120	0.500	0.196
	OsFLN1	0.120	0.450	0.186
	OspTAC7	0.100	0.500	0.180

Interface predicted template modeling (ipTM) and predicted template modeling (pTM) scores were obtained using AlphaFold-Multimer (AFM) analysis (Abramson et al., 2024; https://alphafoldserver.com/welcome). The AFM model confidence score was calculated as 0.8 × ipTM + 0.2 × pTM, with higher scores indicating more reliable predictions for the indicated protein pairs (Homma et al., 2023). OsIAA1–OsARF25 and OsJAZ9–OsSLR1 were included as positive controls.

Table S3. Primers used in this study.

Name	Sequence	Purpose
OsFeSOD3E1_For	CAGAGGTGGTGGATAGATAG	*Cas9-osfesod3E1* genotype
OsFeSOD3E1_Rev	GACTTACCGTGGTGAATACC	*Cas9-osfesod3E1* genotype
OsFeSOD3E4_For	TCCTGATAGCTTCAGTGTAC	*Cas9-osfesod3E4* genotype
OsFeSOD3E4_Rev	GGAAGTCAGATTGAACCAAC	*Cas9-osfesod3E4* genotype
BP_OsFeSOD3_For	GGGGACAAGTTTGTACAAAAAAGCAGGCTATGGCGGCTTTCGCCTCCGC	OsFeSOD3 BP reaction
BP_OsFeSOD3_Rev	GGGGACCACTTTGTACAAGAAAGCTGGGTTCATGCAACTGGGATATTTG	OsFeSOD3 BP reaction
OsFeSOD3-GFP_For	CCCTCTCCCCTTGCTCCGTGGATCCATGGCGGCTTTCGCCTCCGC	35S::OsFeSOD3-GFP construction
OsFeSOD3-GFP_Rev	CCTCGCCCTTGCTCACCATAGGCCTTGCAACTGGGATATTTG	35S::OsFeSOD3-GFP construction
AtPEND cloning_For	CCCCTTGCTCCGTGGATCCATGCACTCTCTTAAGACTACTTGC	pHBT AtPEND-CFP construction
AtPEND cloning_Rev	CCTCGCCCTTGCTCACGGATCCACCAGGACCAAGGACTC	pHBT AtPEND-CFP construction
OsFeSOD3-bait_For	GCCATGGAGGCCGAATTCCCGATGGCGGCTTTCGCCTCCGC	Yeast two hybrid
OsFeSOD3-bait_Rev	CTGCAGGTCGACGGATCCCCTCATGCAACTGGGATATTTG	Yeast two hybrid
OspTAC10-prey_For	GAGGCCAGTGAATTCCACCCGATGGCGGCCACTCCGGCCAC	Yeast two hybrid
OspTAC10-prey_Rev	TCCCGTATCGATGCCCACCCTCATTTTGTGATGAAACACC	Yeast two hybrid
OsFeSOD2-prey_F	AGGCCAGTGAATTCCACCCGATGGCGTTCGCCACACTGGT	Yeast two hybrid
OsFeSOD2-prey_R	TCCCGTATCGATGCCCACCCTCACACCCCTAGGGACTTCT	Yeast two hybrid
OspTAC7-prey_F	GCCATGGAGGCCGAATTCCCCATGGCTATGGCAATGGCGGC	Yeast two hybrid
OspTAC7-prey_R	CTGCAGGTCGACGGATCCCCTCAGTGCCCTTCTTCTTTAAT	Yeast two hybrid
GST-OsFeSOD3_For	GGGGACAAGTTTGTACAAAAAAGCAGGCTCCATGGCGGCTTTCGCCTCCGC	GST fusion
GST-OsFeSOD3_Rev	GGGGACCACTTTGTACAAGAAAGCTGGGTGTGCAACTGGGATATTTGGTTC	GST fusion
MBP-OspTAC10_For	GGGGACAAGTTTGTACAAAAAAGCAGGCTCCATGGCGGCCACTCCGGCCAC	MBP fusion
MBP-OspTAC10_Rev	GGGGACCACTTTGTACAAGAAAGCTGGGTCTTTTGTGATGAAACACCGAT	MBP fusion
MBP-OsFeSOD2_For	GGGGACAAGTTTGTACAAAAAAGCAGGCTCCATGGCGTTCGCCACACTGGTG	MBP fusion
MBP-OsFeSOD2_Rev	GGGGACCACTTTGTACAAGAAAGCTGGGTCCACCCCTAGGGACTTCTCTTG	MBP fusion
MBP-OspTAC7_For	GGGGACAAGTTTGTACAAAAAAGCAGGCTCCATGGCTATGGCAATGGCGGC	MBP fusion
MBP-OspTAC7_Rev	GGGGACCACTTTGTACAAGAAAGCTGGGTCTCAGTGCCCTTCTTCTTTAA	MBP fusion
RT-qPCR_OsFeSOD3_For	GGAAGAGTTTATACGCTCAG	RT-qPCR
RT-qPCR_OsFeSOD3_Rev	TGGACTGATGGCATTTTGTG	RT-qPCR
RT-qPCR_OsRbcL_For	CTCGCGGTATCTTTTTCACTCA	RT-qPCR
RT-qPCR_OsRbcL_Rev	TCGGTCAGAGCTGGCATATG	RT-qPCR
RT-qPCR_OsPsbA_For	CTTGGGCTGATATCATCAAC	RT-qPCR
RT-qPCR_OsPsbA_Rev	GGAACTTCAAGAGCAGCTAG	RT-qPCR
RT-qPCR_OsPsbB_For	ACTTTCCAGTAGTATCGCTG	RT-qPCR

RT-qPCR_OsPsbB_Rev	GGCTAACCCATCACTAACTC	RT-qPCR
RT-qPCR_OsPetD_For	CCGTCAATGATTGGTGAAC	RT-qPCR
RT-qPCR_OsPetD_Rev	CAGAAACCATTAAGAGAACGC	RT-qPCR
RT-qPCR_OsTUB2_For	GAGTTCGACGATGGTGACG	RT-qPCR
RT-qPCR_OsTUB2_Rev	CCACACGGACAGATCATAGG	RT-qPCR
RT-qPCR_OsActin_For	CACTATGTTCCCTGGCATTG	RT-qPCR
RT-qPCR_OsActin_Rev	CTGTACTTCCTTTCAGGAGG	RT-qPCR
